# EpiScanpy: integrated single-cell epigenomic analysis

**DOI:** 10.1038/s41467-021-25131-3

**Published:** 2021-09-01

**Authors:** Anna Danese, Maria L. Richter, Kridsadakorn Chaichoompu, David S. Fischer, Fabian J. Theis, Maria Colomé-Tatché

**Affiliations:** 1 Institute of Computational Biology, Helmholtz Zentrum München, German Research Center for Environmental Health, Neuherberg, Germany; 2 TUM School of Life Sciences Weihenstephan, Technical University of Munich, Freising, Germany; 3 Department of Mathematics, Technical University of Munich, Garching, Germany; 4 Biomedical Center (BMC), Physiological Chemistry, Faculty of Medicine, LMU Munich, Planegg-Martinsried, Germany

**Keywords:** Chromatin analysis, Methylation analysis, Software

## Abstract

EpiScanpy is a toolkit for the analysis of single-cell epigenomic data, namely single-cell DNA methylation and single-cell ATAC-seq data. To address the modality specific challenges from epigenomics data, epiScanpy quantifies the epigenome using multiple feature space constructions and builds a nearest neighbour graph using epigenomic distance between cells. EpiScanpy makes the many existing scRNA-seq workflows from scanpy available to large-scale single-cell data from other -omics modalities, including methods for common clustering, dimension reduction, cell type identification and trajectory learning techniques, as well as an atlas integration tool for scATAC-seq datasets. The toolkit also features numerous useful downstream functions, such as differential methylation and differential openness calling, mapping epigenomic features of interest to their nearest gene, or constructing gene activity matrices using chromatin openness. We successfully benchmark epiScanpy against other scATAC-seq analysis tools and show its outperformance at discriminating cell types.

## Abstract (web summary)

The authors present epiScanpy: a computational framework for the analysis of single-cell epigenomic data, both ATAC-seq and DNA methylation data, with examples for clustering, cell type identification, trajectory learning and atlas integration - and show its performance in distinguishing cell types.

## Introduction

Epigenetic single-cell measurements, where the epigenetic status of single cells is evaluated using next generation sequencing techniques, are becoming mainstream. Currently, two such measurements are performed routinely in the laboratory: DNA methylation can be assessed at the single-cell level with the use of bisulfite sequencing^1^, and open chromatin patterns are investigated at individual cells using Assay for Transposase-Accessible Chromatin using sequencing (scATAC-seq)^2^. Thanks to well described protocols and advances in microfluidics techniques, current experimental designs afford to interrogate the epigenome of thousands of cells at a time^3–7^. These data represent a rich layer of regulatory information that stands between the genome and the transcriptome, and new analysis methods are needed to leverage it.

While many tools for analysing single-cell transcriptomics data exist^8^, fewer are available for scATAC-seq^9–11^, and even less for single-cell DNA methylation data^12,13^. For scATAC-seq data analysis, cisTopic^14^ is the only tool that does both clustering of cells and of open peaks, using Latent Dirichlet allocation (LDA). scABC^15^ and scasat^16^ are methods that use the reads that map into peaks for grouping the cells with k-medoids clustering. Other tools rather consider openness of certain sequence features (like transcription factor motifs^17^, TSSs^18^ or k-mer motifs^19^) to assign cells into groups. The snapATAC^20^ method instead considers the whole genomic information, splitting the genome in equally sized windows, to perform clustering of cells. Meanwhile, other tools are not particularly focused on cell clustering: Cicero^21^ has been developed as a tool to predict cell-type regulatory landscapes given co-accessibility profiles, while Seurat-v3^22^ allows for the anchoring between scATAC-seq and scRNA-seq datasets prior to conversion of the scATAC-seq into a putative gene expression matrix (the so-called gene activity matrix). For single-cell DNA methylation data, only a couple of dedicated analysis tools exist. They mainly aim at the imputation of missing data^12,23^ or at Bayesian clustering of single cells^12^.

In this paper, we present epiScanpy, a method for the analysis of scATAC-seq and single-cell DNA methylation data, which integrates into the scanpy platform for single-cell transcriptomics data analysis^24^. EpiScanpy is therefore the only available tool that offers all analysis options for both scATAC-seq data, single-cell DNA methylation data, as well as scRNA-seq data (via scanpy); and since it builds on scanpy, it makes the full model-zoo of machine learning methods developed for single-cell RNA-seq available to single-cell epigenomics data.

EpiScanpy enables pre-processing of epigenomic data and building of count matrices considering any genomic feature of interest, from open chromatin peaks to whole genome (i.e., windows), as well as any genomic annotation provided as a coordinate or .bed file (genes, enhancers, TFBS, promoters, etc.). Using these constructed count matrices, epiScanpy performs quality control and different downstream analyses such as clustering, marker identification, manifold learning, visualisation and lineage estimation. To take advantage of the multiple scATAC-seq datasets that are currently being generated, epiScanpy also features a function for integration of single-cell open chromatin atlases generated by different laboratories or using different technologies. We have benchmarked epiScanpy to other scATAC-seq tools at their ability to cluster cell types, using four different datasets, and found that epiScanpy is overall superior to them. EpiScanpy works with a flexible data structure, the so-called AnnData^24^, making it a general-purpose platform for future single-cell multi-omics data integration. Since its downstream analyses extend the popular scanpy framework, it inherits properties such as fast and scalable runtime behaviour and modular extensibility.

## Results

### Feature space engineering and data pre-processing

**Fig. 1:**
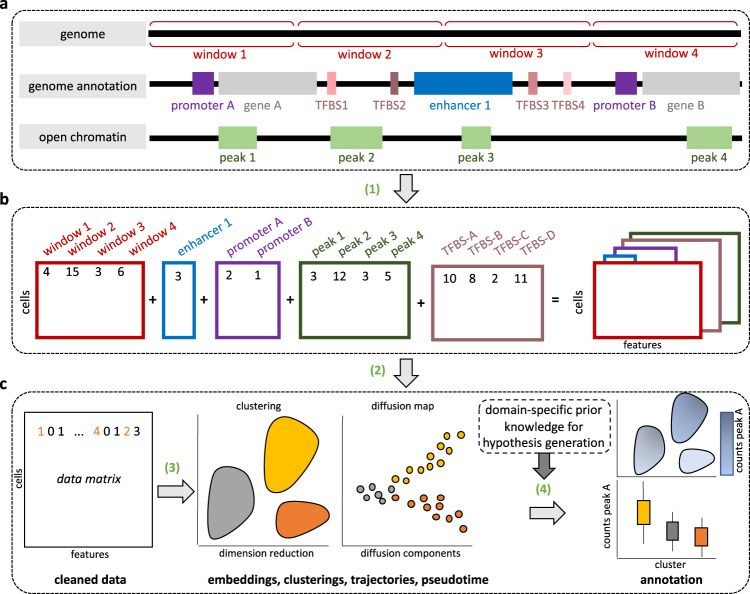
EpiScanpy analysis workflow. **a** epiScanpy quantifies chromatin openness and DNA methylation at different sets of genomic regions to **b** construct count matrices (1) with read counts (for scATAC-seq) or DNA methylation levels (for single-cell DNA methylation). **c** After data pre-processing (2), unsupervised learning algorithms (clusters, trajectories, lineage trees) are applied (3). Differential openness and methylation callings allow for identification of marker loci, which can be used for cell type and lineage tree identification (4).

From .bam files (scATAC-seq) or methylation count files (single-cell DNA methylation), epiScanpy generates count matrices for any genomic features of interest by quantifying the openness or the DNA methylation levels in every feature. These features can cover the entire genome (i.e., windows) or can be based on genomic annotations (such as known open chromatin peaks, gene promoters, enhancers, etc.), or can be any feature coordinates of interest provided as a .bed file (Fig. 1a and Supplementary Methods). To integrate with the often used Chromium Single-Cell ATAC protocol, count matrix construction is also possible from the standard 10x Cell Ranger output, as well as from multiplexed files.

For DNA methylation data, the CG or CH methylation level per feature is calculated as the average methylation level of all the covered cytosines in the feature. For scATAC-seq data, epiScanpy calculates openness summing up all the reads covering a feature. The generated count matrices serve as feature space that retains as much variation of the data as possible without being too high-dimensional—a feature space at single base-pair resolution can in principle be assembled but would impede downstream analysis through memory and runtime issues as well as though data sparsity (Fig. 1b).

After the count matrices have been constructed, epiScanpy proceeds with quality control and data pre-processing (Supplementary methods). For scATAC-seq data, the count matrix is binarised to account for presence/absence of reads at every feature, and library size is normalised. For DNA methylation data, CG or CH methylation level per feature is computed. We differentiate non-methylated features (zero signal) from non-observed features (missing signal) and impute missing data. Note that this is different from imputing zeros in scRNA-seq or scATAC-seq, which are not inherently non-observed data points, but may also be zero count observations. For both single-cell DNA methylation and scATAC-seq data, we discard non-informative features and low-quality cells based on the percentage of cells sharing a feature and the number of covered features per cell (Supplementary Methods and Supplementary Figs. 1 and 2), and select the top most variable features for analysis. EpiScanpy features a series of quality control functions to help the user visualise coverage per cell, as well as coverage and variability per feature, and to select cells and features for downstream analyses (Supplementary methods and Supplementary Figs. 1 and 2).

### Analysis methods for single-cell epigenomic data

**Fig. 2:**
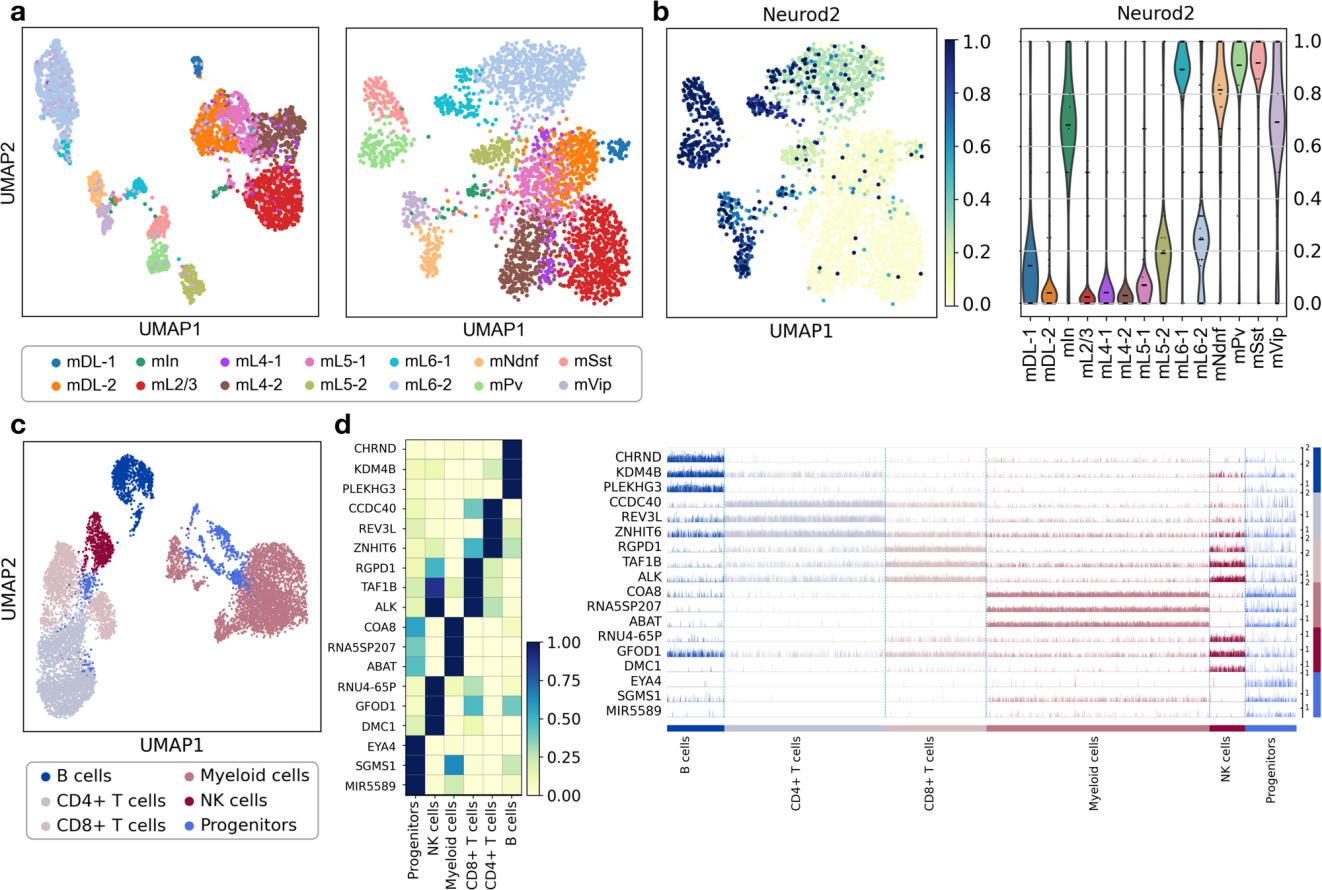
Clustering, visualisation and cell-type annotation for single-cell DNA methylation data and scATAC-seq data. **a** UMAP with annotated cell types for neurons from single-cell DNA methylation data from Luo et al.^3^, performed on the enhancer feature space (left, 3,288 cells x 54,932 enhancers) and promoter feature space (right, 3,224 cells x 32,610 promoters). Annotation: m mouse, DL deep layer, L layer, Ndnf neuron-derived neurotrophic factor, Pv parvalbumin, Sst somatostatin, Vip vasoactive intestinal peptide, In interneurons. **b** UMAP with methylation level at the Neurod2 promoter (a marker of inhibitory neurons) per cell (left) and violin plot with the distribution of Neurod2 promoter methylation per cluster (same colour code as in **a**). Excitatory neurons (mDL-1, mDL-2, mL2/3, mL4-1, mL4-2, mL5-1, mL5-2, mL6-1, mL6-2) have lower methylation at the Neurod2 promoter than inhibitory neurons (mNdnf, mPv, mSst, mVip, mIn). **c** UMAP with annotated cell types for PBMCs from scATAC-seq data from the 10x platform^37^, performed on the open chromatin peak feature space (9,891 cells x 75,226 peaks). **d** Heatmap and track plot indicating openness of the top differential open peaks and their associated genes, which are markers of B cells (CHRND, KDM4B and PLEKHG3, marked in dark blue), T cells (CCDC40, REV3L, ZNHIT6 for CD4+, marked in light grey and RGPD1, TAF1B and ALK for CD8+, marked in light pink), myeloid cells (COA8, RNA5SP207 and ABAT, marked in dark pink), NK cells (RNU4-65P, GFOD1 and DMC1, marked in burgundy) and hematopoietic progenitors (EYA4, SGMS1, and MIR5589, marked in blue). On the heatmap plot, the mean openness per cluster is indicated with a colour scale from 0 (closed) to 1 (open). On the track plot, the openness per cell inside of every cluster is plotted from 0 (closed) to 1 (open). These different cell type identification plots are shown here for DNA methylation (**b**) and ATAC-seq (**d**), but all plots are available for all modalities (Supplementary Figs. 7–9).

After count matrix construction, epiScanpy features the common analysis methods used in single-cell data (Supplementary Methods). In particular, to leverage algorithms that are based on a k-nearest neighbour (kNN) graph, we implement a cell–cell distance metric based on epigenetic features. To assess the global heterogeneity present in the data, epiScanpy uses unsupervised (or manifold) learning algorithms, such as tSNE^25^, UMAP^26^, graph abstraction^27^, Louvain clustering^28^ or diffusion pseudotime^29^ (Fig. 1c). These analyses can be performed on both scATAC-seq and single-cell DNA methylation data (Fig. 2a, c) and using any feature space of interest. To explore unwanted correlations between dataset artefacts (such as coverage) and the variation observed, epiScanpy allows to inspect the relationship between any cell covariate and the principal components (Supplementary Methods and Supplementary Fig. 3). The discovered technical sources of variation can then be regressed out using epiScanpy functions. We also provide a function to optimise the analysis parameters used for Louvain clustering (such as number of PCs and nearest neighbours) based on silhouette scores or adjusted rand index (ARI) (if a cell cluster ground truth is known).

Finally, to determine cell types, epiScanpy includes a differential methylation and differential open chromatin calling strategy, enabling the ranking of genomic features (such as peaks, genes, promoters or other regulatory elements) based on their relevance in the discovered cellular identities (Figs. 1c and 2b, d; and Supplementary methods). This allows to correlate variation across clusters or along trajectories with marker loci to support cell type annotation and to generate hypotheses on the mechanisms that underlie the identified population structure. For scATAC-seq data, epiScanpy also constructs gene activity matrices^21,22^ based on promoter and gene body openness, allowing to call differential gene activity between cell groups. To facilitate cell type annotation, epiScanpy also includes functions to assign any epigenomic feature of interest to its closest gene, or to its closest feature from another single-cell AnnData object (Supplementary Methods). A virtual reality visualisation of epiScanpy’s results can also be done thanks to the virtual reality interface of singlecellVR^30^.

### Applications

We used publicly available scATAC-seq and single-cell DNA methylation datasets to exemplify epiScanpy’s functionalities. For single-cell DNA methylation, we considered a brain dataset with 3,377 prefrontal cortex neurons (4.7% average genomic coverage) from Luo et al.^3^ and built count matrices based on CG methylation levels for different segmentations of the genome: 100 kb non-overlapping windows, promoters, gene bodies and enhancers; as well as CH gene body methylation (Supplementary Fig. 2). The impact of these different genomic feature spaces on the variation retained in the data can be explored using Louvain clustering as an example method for unsupervised learning (Fig. 2a and Supplementary Fig. 4). In general, cells grouped similarly across all feature spaces used, illustrating the fact that different genomic features contain partially redundant information (Supplementary Fig. 4). To quantify clustering results, epiScanpy computes silhouette scores^31^ (Supplementary methods), a measure of how similar a cell is to its own cluster compared to other clusters. Interestingly, the enhancer feature space provided the clearest cell-type separation, with an average silhouette score of 0.41 (average of the silhouette score across all cells), compared to 0.32, 0.28 and 0.09 for windows, promoters and gene bodies (Supplementary Fig. 5). This result highlights the relevance of DNA methylation at non-genic regulatory elements for determining cell identity. To identify cell type labels, epiScanpy features a differential methylation test between clusters (Fig. 2b and Supplementary methods). We ranked the top most differentially methylated promoters per cluster and identified 17 different cell types using known neuronal marker genes (Fig. 2a, b and Supplementary Figs. 6 and  7).

We next used a chromatin accessibility dataset from the 10x platform containing 10,000 PBMC cells (10k Peripheral blood mononuclear cells (PBMCs) from a healthy donor). After calling open chromatin peaks using MACS2^32^ on the pseudo-bulk dataset, we built a peak accessibility count matrix and used it to perform dimensionality reduction and Louvain clustering (Supplementary methods), identifying 6 clusters in the dataset (Fig. 2c). To assign cell identity labels to every cluster, epiScanpy performed differential openness tests, ranking peaks by their differential openness between clusters. To facilitate gene marker identification from the identified differential peaks, epiScanpy features a function to assign every differential peak to its most proximal promoter. We used these most proximal genes to perform broad cell type identification, namely progenitors, B cells, T cells (CD4+ and CD8+), myeloid cells and natural killer (NK) cells (Fig. 2d and Supplementary Fig. 8).

**Fig. 3:**
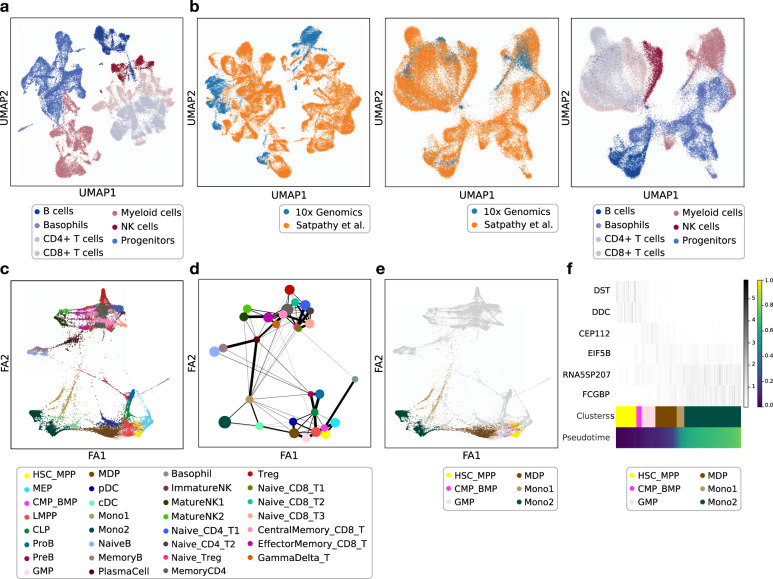
Data integration, partition-based graph abstraction (PAGA) and diffusion pseudotime in scATAC-seq. **a** UMAP with annotated cell types from scATAC-seq for blood cells from Satpathy et al.^5^, performed on the peak feature space (57,177 cells x 83,823 peaks). Only the broad cell type annotation is shown. **b** Joint UMAP for two scATAC-seq datasets with experiment label (10x Genomics and Satpathy et al.) for concatenated count matrices (left) and mixed using BBKNN with experiment label (middle) and cell type label (right) (62,284 cells x 123,280 peaks). **c** Force-directed graph drawing of the Satpathy et al. dataset. **d** PAGA plot for the same cells using the same Force-directed graph embedding. **e** Monocyte differentiation path depicted on top of the force-directed graph drawing, and **f** openness of peaks at marker genes during pseudotime progression (distance) in the monocyte differentiation path (16,004 cells x 83,823 peaks).

EpiScanpy can also be used to integrate scATAC-seq datasets produced by different laboratories and using different experimental protocols, using the batch corrected k-nearest neighbours (BBKNN) algorithm^33^ (Supplementary methods). Other available algorithms for epigenomic data integration are snapATAC^20^ (which uses Harmony^34^) or LIGER^35^ (which uses integrative non-negative matrix factorisation), and other scRNA-seq integration methods can also be applied to scATAC-seq data^36^. To exemplify scATAC-seq data integration, we integrated a chromatin accessibility dataset from Satpathy et al.^5^, featuring 63,882 blood cells (Fig. 3a and Supplementary Fig. 9), to the scATAC-seq PBMC dataset from the chromium 10x platform described above. For the integration, we used the union of the peaks from the two datasets to construct a concatenated open chromatin count matrix (Supplementary Method) and to find the set of common nearest neighbours between the datasets. We generated a joint kNN graph and embedding using the BBKNN algorithm^33^ (Supplementary Information). After integration, the cells from the two different origins were well mixed, and the cell types correctly merged (Fig. 3b). ScATAC-seq atlas integration also works well for other cell types and organs and can be done using other feature spaces. For example, we also integrated two scATAC-seq brain datasets from 10x^37^ and Fang et al.^20^, which contain terminally differentiated neurons, using 5 kb windows as the common feature space (Supplementary Fig. 10).

The Satpathy et al.^5^ dataset contains >60,000 blood cells. Since blood is in continuous differentiation, from hematopoietic stem cells to fully differentiated cells via a variety of intermediate progenitors, it is best characterised by a continuous representation instead of a clustering in fully differentiated cell types. For that, we used epiScanpy to produce continuous representations of that data, using the more detailed cell type annotation from Satpathy et al. (Supplementary Fig. 9). We used partition-based graph abstraction (PAGA)^27^ and Force-directed graph drawing^38^ (Fig. 3c, d) to generate a topology-preserving map of single cells based on their peak openness. PAGA has the ability to preserve both continuous and disconnected structure in the data at multiple resolutions. Finally, diffusion pseudotime can also be utilised in this dataset (Supplementary Fig. 11) as a tool for dimensionality reduction, ordering the cells by changes in peak openness along diffusion components. In all cases, the connections between cell types can be identified, and the most likely differentiation paths can be explored. For example, monocytes originate from hematopoietic stem cells, and after transitioning through multipotent progenitors, common myeloid progenitors, granulocyte-macrophage progenitors, and monocyte-dendritic cell progenitors arrive at two distinct populations of monocytes (Fig. 3e). Along any trajectory, the cells can be ordered according to their diffusion pseudotime, and the peaks that become progressively open and closed can be identified and associated with genes in their proximity and visualised (Fig. 3f and Supplementary Fig. 12).

### EpiScanpy scATAC-seq analysis is benchmarked in comparisons with established packages

**Fig. 4:**
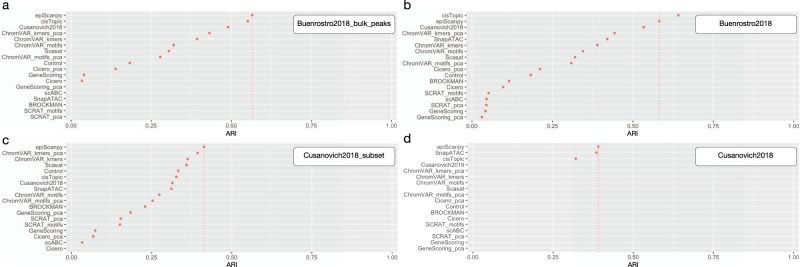
Benchmarking of cell clustering performance. Adjusted rand index (ARI) for Louvain clustering in **a** Buenrostro et al.^40^ dataset for bulk peaks with 2,034 cells, **b** Buenrostro et al.^40^ dataset with 150,429 open features and 2,034 cells, **c** Cusanovich et al.^4^ mouse atlas downsampled to 12,178 cells, **d** full Cusanovich et al.^4^ mouse atlas with 81,173 cells. EpiScanpy performance results are compared to the results of 11 other scATAC-seq methods benchmarked in Chen et al.^11^. The dotted lines indicate epiScanpy’s ARI value.

We have compared epiScanpy’s ability to discriminate cell types to 11 other scATAC-seq data analysis tools, using the framework and results proposed by Chen et al.^11^. We find that epiScanpy scores consistently among the top tools in all the tested datasets and is only outperformed by another method, CisTopic^14^, in one dataset (Fig. 4, Supplementary Fig. 13 and Supplementary Information). Interestingly, compared to epiScanpy, all other top methods assessed have less robust performance across datasets (Fig. 4), performing well in some scenarios but dropping in performance in the other datasets.

An important feature of epiScanpy is its ability to scale to large datasets in a very competitive runtime, for example, analysing the whole mouse scATAC-seq atlas from Cusanovich et al.^4^, consisting of 81,173 cells, in a mere runtime of 18.19 min using 14.19 GB of memory (Supplementary Fig. 13). This ability to scale up to large datasets with such fast runtimes allows for a much needed interactive exploration of large scATAC-seq datasets. Such competitive runtimes and scalability to large datasets is an asset of epiScanpy, which is missing in competing R-based analysis tools because of inherent memory limitations in R. We have compared epiScanpy speed and memory usage to the other top performing method, cisTopic, using CentOS Linux 7, on an AMD Opteron 6376 2.3 GHz machine with 8 cores of CPU, 180GB of memory, and 245/45.2 MB/s of input/output speeds. EpiScanpy consistently outperformed in terms of memory consumption in all datasets and comparably performed in terms of runtime for the smaller datasets while it outperformed for the larger ones (Supplementary Fig. 13).

## Discussion

EpiScanpy is a fast and versatile tool for the analysis of single-cell epigenomic data, and it offers the common framework for the analysis of both single-cell DNA methylation and scATAC-seq data, as well as single-cell transcriptomic data thanks to its embedding in the scanpy platform. Its flexible data structure is ready to handle other new types of single-cell omic data, such as Hi-C or NOME-seq, as well as multi-omics single-cell data. EpiScanpy performs common analysis like low-dimensional data visualisation, clustering, single-cell graph abstraction, trajectory inference, and differential calling, based solely on epigenomic features. It also features a series of useful downstream functions, such as the mapping of epigenomic features of interest to their closest gene, or the construction of gene activity matrices based on promoter openness. It includes an atlas comparison tool that effectively integrates scATAC-seq datasets generated in different laboratories and/or using different platforms. Such chromatin-centric data integration strategies will be necessary to leverage the large number of single-cell open chromatin datasets being generated. EpiScanpy was benchmarked against other 11 scATAC-seq methods, and it consistently scores among the top tools on its ability to discriminate cell types. EpiScanpy is available as an open-source python package through Github (https://github.com/colomemaria/epiScanpy, https://colomemaria.github.io/episcanpy_doc) and is built upon the scanpy analysis toolbox^24^, opening the scRNA-seq toolchain to the commonly measured single-cell epigenomic data.

## Methods

### EpiScanpy DNA methylation count matrix construction

For single-cell DNA methylation, epiScanpy builds count matrices from cytosine summary tables. EpiScanpy can build count matrices for any feature space of interest (for example, a set of genomic regions/annotations inputted as a .bed file, or windows spanning the whole genome), retrieving the methylation status of cytosines in CG, CH or both genomic contexts in every feature. To account for the low cytosine content of certain features (either CG, CH, or both) and to account for the low coverage intrinsic to single-cell DNA methylation data, epiScanpy can filter out features containing too little number of cytosines or too little number of reads. The user can specify the minimum number of cytosines covered per feature to return a methylation level. Then epiScanpy filters out features that are covered in too few cells and cells that do not have enough covered features. After this filtering step, there are features which, for some cells, have still a missing value. These methylation values are then imputed as the average methylation level of the feature across all cells. EpiScanpy can save the data matrix before imputation in a different layer of the AnnData object.

### EpiScanpy ATAC-seq count matrix construction

For scATAC-seq, epiScanpy constructs count matrices starting from multiplexed .bam files and fragment files, such as the 10x Cell Ranger output, or directly from demultiplexed files. EpiScanpy generates count matrices for any genomic annotation of interest (peaks, windows, enhancers, promoters, etc., or any provided annotation as a .bed file) (Fig. 1a and Supplementary Figs. 4, 5, 7). For scATAC-seq data, the number of reads in every feature are added up and then the count matrix is binarised to account for presence/absence of reads at every feature, and library size is normalised. Additional linear regression of covariates is available for both ATAC and DNA methylation data. For scATAC-seq, epiScanpy also calculates gene activity matrices by summing the reads intersecting the promoter (default value: 5000 bp from TSS) and the gene body for every gene^8,21^. To assign epigenomic features such as peaks to their closest genes, epiScanpy features a function that finds either the closest gene to any feature or finds the genes in a given proximity (number of bp to be specified by the user).

### EpiScanpy workflow

Several functions are implemented in epiScanpy to explore the data and perform quality control, to identify the best parameters for discarding low covered cells and low covered genomic features:

- A histogram plot of cell coverage to identify lowly covered cells (Supplementary Figs. 1 and 2).
- A function to filter low-quality cells based on the coverage histogram.
- A histogram plot of feature coverage in the cellular population to identify features which are not covered in enough cells (Supplementary Figs. 1 and 2).
- A function to filter features based on the above coverage histogram (filter based on a number of cells being covered).
- A function to rank features based on their variability in the population of cells. Maximum variable features (variability = 1) are these where half the cells are open and half the cells are closed. Minimum variable features (variability = 0) are these where all cells are closed or all cells are open.
- A function to select the most variable features based on the ranking of feature variability, top variable features are selected either as a percentage of features to retain or as a number of features to retain (Supplementary Fig. 1).
- A plot of any cell covariate (stored in AnnData.obs) versus any principal component (PC). This plot is made specially to explore the existence of a correlation between cell total coverage and the PC of interest (by default PC1), which is an indication that library size per cell needs to be normalised (Supplementary Fig. 3).
- A plot to show the variance ratio per principal component to guide the selection of the number of PCs to retain for the analysis (Supplementary Fig. 3).

After quality control and filtering, the count matrix (cells times features) is normalised to account for differences in library size and/or technical artefacts using count per million normalisations and/or linear regressions. The normalised matrix is then used to calculate a cell–cell distance metric based on Euclidean distance between the epigenomes of pairs of cells and to construct a k-nearest neighbour (knn) graph. Afterwards, common algorithms that use that knn graph can be applied, such as Louvain clustering^28^, diffusion pseudotime^29^ and UMAP^26^. Other unsupervised learning algorithms, such as tSNE^25^ and graph abstraction^27^ can also be used.

EpiScanpy provides multiple functions to explore the best analysis parameters (such as the number of PCs to consider, number *k* of nearest neighbours) to optimise the best cell clustering possible. To this end, epiScanpy offers multiple clustering functions such as hierarchical clustering, kmeans, Louvain and Leiden. The validity and relevance of the resulting clusters can be explored further using different metrics available in epiScanpy like silhouette scores (wrapper of scikit-learn function). Sometimes a ground truth (cell type) is also known. In these cases, epiScanpy can also calculate the adjusted rand index (ARI), Adjusted Mutual Information (AMI) and a homogeneity metric using the known cell identity (wrapper of scikit-learn functions).

To identify differential features between cell groups, we take advantage of the large cell number and use logistic regression on the epigenomic levels of features between groups (whether these groups are defined by Louvain clusters or by experimental cell type annotations or any other grouping of interest), following Ntranos et al.^39^. EpiScanpy outputs a list of ranked features with the results of the differential test, that the user can utilise for downstream analysis.

If the user has several count matrices for the same organism, organ or tissue, that need to be compared (for example, to compare -omics layers, where there is one AnnData object per layer), the user can upload the different count matrices at the same time. After pre-processing of every matrix separately, epiScanpy has functions to identify the closest features between count matrices. For example, if one count matrix contains genes and the other one epigenomic features such as peaks, epiScanpy identifies the closest gene to every epigenomic feature, given a search size specified by the user (by default 5000 bp around the epigenomic feature). The user can also focus on a set of interesting features, for example, a list of differentially open peaks in the scATAC-seq dataset, and match the coordinates of every one of them to its closest gene from the gene expression count matrix, or its closest methylation locus from the single-cell DNA methylation count matrix. Functions like label_transfer, transfer_obs or transfer_var help to compare different -omics, datasets and feature spaces. If the interest is, for example, in differential features, a comparison of features between -omics will reveal which ones are differentially open + differentially expressed + differentially methylated between -omic layers, versus features that are differential in only one -omic layer but non-differential in the other ones

### EpiScanpy chromatin data integration workflow

In the advent of having multiple datasets of the same omic (single-cell ATAC-seq or DNA methylation) to analyse jointly, it is important to remove potential batch effects. EpiScanpy offers this possibility using the bbKNN^33^ batch correction method. In order to integrate the different batches, it is required to use a common feature space. Thus, a preliminary step is to build count matrices using a shared set of features like windows or a common set of peaks between datasets. To obtain a good embedding of the different datasets together, it is important that the set of features used is representative of all datasets. For that, we select the most variable features on each dataset separately. Then we concatenate the datasets keeping the intersect of the variable features. Alternatively, epiScanpy can merge the datasets using the union of the different feature spaces. Additional quality controls and filtering are recommended to remove features that are not covered in enough cells, and cells which do not contain enough covered features. Finally, we proceed to library size normalisation and run the integration method on this concatenated matrix.

### Reporting summary

Further information on research design is available in the Nature Research Reporting Summary linked to this article.

## Supplementary information

Supplementary Information

Reporting Summary

## Data Availability

The following publically available datasets analysed in this study can be downloaded from the GEO with accession codes GSE129785 and GSE97179. Access to the genome annotation used: mm10 [ftp://hgdownload.soe.ucsc.edu/goldenPath/mm10/]. All pre-processed data used in the paper can be accessed in the Zenodo platform with the identifier 10.5281/zenodo.4292082.

## Code availability

EpiScanpy is available through Github (https://github.com/colomemaria/epiScanpy) and the documentation is available at https://colomemaria.github.io/episcanpy_doc. All code used for the analysis of the data, as well as for the benchmarking, is available at https://github.com/colomemaria/episcanpy-paper (10.5281/zenodo.4564324).
