# Supplementary material for: EpiScanpy: integrated single-cell epigenomic analysis
Source: Nat Commun. 2021 Sep 1;12:5228. doi: 10.1038/s41467-021-25131-3 (PMC8410937; doi:10.1038/s41467-021-25131-3)
Supplement: Supplementary file 1 — Supplementary Information [file 41467_2021_25131_MOESM1_ESM.pdf]

Supplementary Information Appendix

EpiScanpy: integrated single-cell epigenomic analysis

Authors: Anna Danese[1], Maria L. Richter[1], Kridsadakorn Chaichoompu[1], David S. Fischer[1,3], Fabian J. Theis[1,2,3]*, Maria Colomé-Tatché[1,2]*

Affiliations:

1 Institute of Computational Biology, Helmholtz Center Munich, German Research Center for Environmental Health, Neuherberg, Germany

2 TUM School of Life Sciences Weihenstephan, Technical University of Munich, Freising, Germany

3 Department of Mathematics, Technical University of Munich, Garching, Germany

* correspondence to:

fabian.theis@helmholtz-muenchen.de and maria.colome@helmholtz-muenchen.de

Contents :

EPISCANPY WORKFLOW 3

USED DATASETS 4

GENOME ANNOTATION 5

Luo et al. BRAIN DNA methylation (snmC-seq) ANALYSIS 6

10x PBMC scATAC-seq ANALYSIS 7

Satpathy et al. whole blood scATAC-seq ANALYSIS 7

INTEGRATION OF PBMC scATAC-seq DATASETS 8

INTEGRATION OF BRAIN scATAC-seq DATASETS 9

BENCHMARKING TO OTHER scATAC-seq METHODS 9

CODE AVAILABILITY 10

SUPPLEMENTARY FIGURES 12

Supplementary methods EPISCANPY WORKFLOW

For scATAC-seq, epiScanpy constructs count matrices starting from multiplex .bam files and fragment files, such as the 10x Cell Ranger output, or directly from demultiplexed files. For DNA methylation data, epiScanpy constructs count matrices from methylation count files (single-cell DNA methylation). EpiScanpy generates count matrices for any genomic annotation of interest (peaks, windows, enhancers, promoters, etc, or any provided annotation as a .bed file) (Fig. 1a, and Supplementary Fig. 4-5, 7).

For scATAC-seq data, the number of reads in every feature are added up and then the count matrix is binarized to account for presence/absence of reads at every feature, and library size is normalized. For DNA methylation data, CG or CH methylation level per feature is computed as the average DNA methylation in the cytosines present in the feature. Additional linear regression of covariate is available for both ATAC and DNA methylation data. For scATAC-seq, epiScanpy also calculates gene activity matrices by summing the reads intersecting the promoter (default value: 5000bp from TSS) and the gene body for every gene[1,2].

To assign epigenomic features such as peaks to their closest genes, epiScanpy features a function that finds either the closest gene to any feature, or finds the genes in a given proximity (number of bp to be specified by the user).

Several functions are implemented in epiScanpy to explore the data and perform quality control, to identify the best parameters for discarding low covered cells and low covered genomic features:

-histogram plot of cell coverage, to identify lowly covered cells (Supplementary Fig. 1 and S2),

-function to filter low quality cells, based on the coverage histogram above,

-histogram plot of feature coverage in the cellular population, to identify features which are not covered in enough cells (Supplementary Fig. 1-2),

-function to filter features based on the above coverage histogram (filter based on a number of cells being covered),

-function to rank features based on their variability in the population of cells (maximum variable features (variability = 1) are these where half the cells are open and half the cells are closed, minimum variable features (variability = 0) are these where all cells are closed or all cells are open)

-function to select the most variable features (based on the ranking of feature variability, top variable features are selected either as a percentage of features to retain or as a number of features to retain) (Supplementary Fig. 1),

-plot of any cell covariate (stored in annData.obs) versus any principal component (PC). This plot is made especially to explore the existence of a correlation between cell total coverage and the PC of interest (by default PC1) which is an indication that library size per cell needs to be normalized (Supplementary Fig. 3),

-plot showing the variance ratio per principal component, to guide the selection of the number of PCs to retain for the analysis (Supplementary Fig. 3).

After quality control and filtering, the count matrix (cells times features) is normalized to account for differences in library size and/or technical artefacts using count per million normalization and/or linear regression. The normalized matrix is then used to calculate a cell-cell distance metric based on Euclidean distance between the epigenomes of pairs of cells and to construct a k-nearest neighbor (knn) graph. Afterwards, common algorithms that use that knn graph can be applied, such as Louvain clustering[3], diffusion pseudotime[4] and UMAP[5]. Other unsupervised learning algorithms, such as tSNE[6] and graph abstraction[7] can also be used. EpiScanpy provides a function to explore the best analysis parameters (such as number of PCs to consider, number k of nearest neighbours) to optimize the Louvain clustering results using silhouette scores (using the silhouette function from scikit-learn). Sometimes a ground truth (cell type) is known. In these cases, epiScanpy also calculates the adjusted rand index (ARI) using the known cell identity (function from scikit-learn).

To identify differential features between cell groups, we take advantage of the large cell number and use logistic regression on the epigenomic levels of features between groups (whether these groups are defined by Louvain clusters or by experimental cell type annotations or any other grouping of interest), following Ntranos et al.[8]. EpiScanpy outputs a list of ranked features with the results of the differential test, that the user can utilize for downstream analysis.

If the user has several count matrices for the same organism, organ or tissue, that need to be compared (for example, to compare -omics layers, where there is one AnnData object per layer), the user can upload the different count matrices at the same time. After pre-processing of every matrix separately, epiScanpy has functions to identify the closest features between count matrices. For example, if one count matrix contains genes and the other one epigenomic features such as peaks, epiScanpy identifies the closest gene to every epigenomic feature, given a search size specified by the user (by default 5000bp around the epigenomic feature). The user can also focus on a set of interesting features, for example a list of differentially open peaks in the scATAC-seq dataset, and match the coordinates of every one of them to its closest gene from the gene expression count matrix, or its closest methylation locus from the single-cell DNA methylation count matrix. Functions like label_transfer, transfer_obs or transfer_var help to compare different -omics, datasets and feature spaces. If the interest is for example in differential features, a comparison of features between -omics will reveal which ones are differentially open + differentially expressed + differentially methylated between -omic layers, versus features which are differential in only one -omic layer but non-differential in the other ones.

USED DATASETS

To provide examples of the epiScanpy functionalities, we used different single-cell (sc) DNA methylation and scATAC-seq datasets. These are:

-methylation profiles from Luo et al.[9], GEO: "GSE97179 [https://www.ncbi.nlm.nih.gov/geo/query/acc.cgi?acc= GSE97179]".This sc DNA methylation dataset was obtained applying single-nucleus methylcytosine sequencing (snmC-seq) to 3377 prefrontal cortex neurons (NeuN+) of 8-week old mice, yielding 4.7% average coverage of the mouse genome per single cell. The different neuron subtypes were not labelled experimentally. The downloaded datasets contain cytosine summaries for all cells with columns for genomic position, number of methylated cytosines, number of total cytosines, and cytosine context (CG, CH; where H=A,T,C). The sequencing data were aligned on GRCm38, all processing specifications and metadata are available as supplementary material in Luo et al.[9].

-brain chromatin accessibility datasets from Fang et al.[10] sci-ATAC (replicate 312_3B), downloaded from http://renlab.sdsc.edu/r3fang/share/

-brain 10x[11] datasets, they were downloaded from http://renlab.sdsc.edu/r3fang/share/ as 5kb size windows count matrix.

- scATAC-seq 10x PBMC 10k cells, obtained using the 10x chemistry Next Gem v1.1, and analysed using cellranger v1.2.0. The fragment files downloaded had been aligned on hg19. Data available here: https://cf.10xgenomics.com/samples/cell-atac/1.2.0/atac_pbmc_10k_nextgem/

-Satpathy et al.[12] whole blood fresh data: The raw peak count matrix as well as the fragment files from the corresponding samples were downloaded from GEO: "GSE129785 [https://www.ncbi.nlm.nih.gov/geo/query/acc.cgi?acc=GSE129785]". The raw count matrix (GSE129785_scATAC-Hematopoiesis-All.mtx.gz) contains 63882 cells × 571400 peaks.

-Buenrostro et al. 2018[13] PBMC scATACseq, and Cusanovich et al. 2018[14]: these datasets were used for the benchmarking of epiScanpy using the framework from Chen et al.[15]. They were downloaded from https://github.com/pinellolab/scATAC-benchmarking.

GENOME ANNOTATION

Promoter annotations: TSS positions from the Eukaryotic Promoter Database[16] were used, and promoters were defined as -1.5kb upstream of the TSS and +500 bp downstream.

Enhancer annotations: enhancer annotation was downloaded from the Mouse Encode Project at Ren Lab[17] (http://chromosome.sdsc.edu/mouse/download.html). Enhancers are 2000 bp long. The mm9 annotations were converted to mm10 using LiftOver[18].

Gene body annotations: gene bodies from UCSC ("mm10 [ftp://hgdownload.soe.ucsc.edu/goldenPath/mm10/]") were used.

Peaks: peaks used to analyse the Satpathy et al.[12] data are available from GEO "GSE129785 [https://www.ncbi.nlm.nih.gov/geo/query/acc.cgi?acc=GSE129785]". Peaks used to build the count matrices for 10x 10k PBMC data were called using MACS2[19]. A merge set of peaks was used to integrate the Satpathy et al. and the 10x 10k PBMC data, using the union of the aforementioned peaks.

Luo et al. BRAIN DNA METHYLATION ANALYSIS

Starting from the methylation summary files obtained from Luo et al.[9], we built count matrices based on different segmentations of the genome. We used epiScanpy to build count matrices with 100kb non-overlapping windows, promoters, gene bodies and enhancers (Supplementary Fig. 2).

For each feature and each cell of the count matrix, we computed the average methylation level based on the number of methylated and unmethylated reads of cytosines either in a CG or CH (H=A, T, C) context. For large genomic regions such as 100kb windows, we set a minimum number of cytosines (in CG or CH context) to be covered in order to calculate an average methylation level. If these minima are not reached, a 'NA' (Not Available) is added in the matrix. For smaller genomic regions such as promoters or enhancers, the minimum number of covered cytosines (in CG or CH context) is set to one, otherwise, the feature is labelled as missing data ('NA').

The raw count matrices contain a large number of missing values. We filtered out features shared in less than a 1000 cells and removed cells with less than a 1000 features covered.

Since the single-cell DNA methylation dataset from Luo et al. has a genomic coverage of ~4.7% on average[9], the obtained methylation count matrices are very sparse, containing many missing values that correspond to uncovered cytosines and which are represented as 'NA'. Depending on the size of the features considered to build the count matrix, as well as the minimum coverage threshold required, the distribution of the missing values change drastically (Supplementary Fig. 2). In order to perform dimensionality reduction, it is necessary to filter features which are not covered in the majority of cells, and for the remaining ones it is necessary to impute missing information in the uncovered cells, otherwise, PCs cannot be computed. For imputation, we replace the missing values by the mean value of that feature in the population of cells (after filtering). After clustering, it is possible to refine the imputed value by assigning the average methylation level of the cells within a cluster.

For all imputed count matrices corresponding to the different annotations, we computed principal components and ratio of explained variances. Then we built a neighborhood graph using Euclidean distance. For low dimensional representation, we used principal component analysis (PCA) and uniform manifold approximation and projection (UMAP)[5] (Fig. 2a and Supplementary Fig. 4). t-distributed stochastic neighborhood embedding (t-SNE) can also be performed using epiScanpy.

We used Louvain clustering on every feature matrix independently (i.e. windows, promoters, enhancers and gene bodies), to identify initially two main clusters, corresponding to excitatory and inhibitory neurons, and obtained the most differentially methylated features between them. Using the promoter count matrix for cell type identification, we obtained the top 400 most differentially methylated promoters between the two clusters. Among the top results, we identified promoters of known marker genes for both excitatory and inhibitory neurons and labelled the clusters accordingly. We repeated the clustering and cell type identification iteratively treating inhibitory and excitatory neurons independently, by dividing the matrix into two submatrices (Fig. 2a, Fig 2b, and Fig S6). We

also used CH gene body methylation of known marker genes as a validation of the obtained promoter CG methylation-based cell type identification (Supplementary Fig. 6-7).

We explored the impact of the choice of genomic feature space on the global topology ("structure") that can be learned from the data, using clustering as an example method for unsupervised learning. Cells grouped similarly across all feature spaces used, illustrating the fact that different genomic features contain partially redundant information (Supplementary Fig. 4). We computed silhouette scores for promoter- and enhancer-based analysis using the scikit-learn function silhouette[20], which has been implemented as part of epiScanpy. Interestingly, the enhancer feature space provided the clearest cell-type separation in clustering and low dimensional visualization (average silhouette score of 0.44 (enhancers) versus 0.36 (promoters)), highlighting the relevance of DNA methylation at non-genic regulatory elements for determining cell identity (Supplementary Fig. 5).

10x PBMC scATAC-seq ANALYSIS

To use the 10x scATAC-seq data, we first performed de-novo open chromatin peak calling. The peaks were called from the fragment file using MACS2[19]. Then we built and processed the peak matrix using epiScanpy. The matrix was first binarized and empty barcodes and peaks were removed. The cell barcodes were filtered if they contained less than 100 peaks covered, while the peaks covered in less than 10 cell barcodes were removed as well. Then, we performed quality controls to further remove low quality cells (cells with less than 1200 peaks covered), low quality peaks (covered in less than 20 cells) and to select the most variable peaks (variability score above 0.5139) (Supplementary Fig. 1). Finally, we normalised the matrix to correct for library size and log transformed. Thus, we obtained a matrix containing 9891 cells and 75226 peaks.

To build the Uniform Manifold Approximation and Projection for Dimension Reduction (UMAP) embedding we calculated the first 50 principal components (PCs) and the neighborhood graph with the 50 PCs and the 15 nearest neighbors. We clustered the cells using Louvain clustering and identified the top differential peaks (Fig. 2c-d, and Supplementary Fig. 8). To establish a preliminary cell type annotation of the clusters, we considered the differential peaks linked to genes (peaks overlapping the gene body or up to 5kb upstream of the TSS) as well as the top differential genes using a gene activity count matrix. We built the gene activity matrix, from the peak count layer, using the number of open peaks overlapping both the promoter (5k upstream of the TSS) and the gene body. The matrix is normalised according to the peak library size and while keeping the peak embedding, we call the top 200 differential genes between the different Louvain clusters. We later confirmed the cell type annotation using cell label transfer with the Satpathy et al.[12] whole blood scATAC-seq annotated dataset.

Satpathy et al. WHOLE BLOOD scATAC-seq ANALYSIS

The initial matrix of 63882 cells × 571400 peaks was binarized and cells with fewer than 2000 peaks were removed. Peaks that were covered in less than 50 cells were removed and selected the most

variable peaks (Supplementary Fig. 1). This resulted in a matrix of 57177 cells x 83823 peaks. Normalization was done for each cell based on the total counts over all features. Cluster annotation was taken from Satpathy et al.[12]. Dimensionality of the normalized matrix was reduced by calculating the first 50 PCs. Based on these PCs, a neighborhood graph was constructed based on 15 nearest neighbors, where the connectivities between data points are calculated using UMAP. UMAP was also used for embedding the data points for visualization.

To make sense of the peaks, the region within 5kb upstream of every peak was searched for genes. For every cell type, the top-ranked features were searched for peaks in proximity of genes (Supplementary Fig. 9). Partition-based graph abstraction (PAGA) was used to generate a topology-preserving map of single cells based on their peak openness (Fig. 3c,d). These calculations were then further used to construct a PAGA-initialized embedding of the cells. Additionally, diffusion pseudotime (dpt) was used as another tool for dimensionality reduction, ordering the cells by changes in peak openness along diffusion components (Supplementary Fig. 11). Per cell type, one of the top-ranked open peaks that appeared to be within 5000bp upstream of a gene was taken to visualize changes in openness along dpt in a PAGA path. This was done individually for several hematopoietic lineages, and the peaks were annotated by their nearest gene.

INTEGRATION OF PBMC scATAC-seq DATASETS

In order to merge the two datasets we first defined a new set of features as the union of the peaks called for the 10x PBMC dataset and the peaks from Satpathy et al.[12] whole blood data. We built the corresponding count matrices for 10x PBMC and Satpathy et al. from their respective fragment files. The preliminary processing and quality controls of the two count matrices was done separately.

The raw 10x count matrix was first binarised, cell barcode with less than 800 open peaks and peaks open in less 15 cells were filtered out. We obtained 10234 cells passing the quality controls. Then we reduced the feature space to the top 150513 most variable peaks, normalised per library size and log transform the count matrix.

The Satpathy et al. whole blood data contain several samples (GSM3722027 monocytes, GSM3722028 B cells, GSM3722029 CD34+ progenitors rep1, GSM3722030 Regulatory T cells, GSM3722031 Naive CD4+ T cells rep1, GSM3722032 memory CD4+ T cells rep1, GSM3722033 CD4+ helper T cells, GSM3722036 NK cells, GSM3722037 naive CD8+ T cells, GSM3722038 memory CD8+ T cells, GSM3722071 bone marrow rep1, GSM3722072 CD34+ progenitors rep2, GSM3722073 memory CD4+ T cells rep2, GSM3722074 naive CD4+ T cells rep2, GSM3722075 PBMC rep3, GSM3722076 PBMC rep2, GSM3722077 PBMC rep4), we built count matrices for each sample, filtered all the cell barcodes containing less than 100 open peaks and concatenated the samples, using epiScanpy.

Once we obtained the raw count matrix containing all the samples from Satpathy et al., we binarized the matrix, performed quality controls, further removed cell barcodes containing less than 600 open features, un-annotated cells and peaks open in less than 20 cells. We obtained 52050 cells passing the

quality controls. Then we selected the top 150051 most variable peaks, normalised per library size and log transform the count matrix.

Once both datasets were filtered and normalised, we concatenated the count matrices and obtained 62284 cells with 123280 peaks. We built the knn graph using the first 50 PCs and 15 neighbors, then we constructed the UMAP embedding showing a batch effect between the two datasets (Fig. 3b) that we corrected using bbknn[21] (Fig. 3b).

INTEGRATION OF BRAIN scATAC-seq DATASETS

The Fang et al.[10] sci-ATAC (replicate 312_3B) and the brain 10x[11] datasets were downloaded from http://renlab.sdsc.edu/r3fang/share/. To show how different feature spaces can be used for integration, here for integration we considered 5kb size windows. For the 10x dataset we considered cells that have coverage in at least 500 windows, and windows are kept if they are covered in at least 100 cells, obtaining a matrix with 4349 cells x 164259 windows. For the sci-ATAC data, we considered windows overlapping the reduced 10x dataset, and we removed cells with less than 500 windows covered. Then we concatenated the two datasets, and we removed windows with less than 100 cells covered per dataset, and further removed windows that covered less than 500 cells across the 2 datasets, obtaining a matrix with 13189 cells x 54539 windows. Finally, we regressed out the cells based on the number of windows covered.

We first performed Louvain clustering in the two datasets independently, and then we built a joint neighbouring graph using bb-knn[21]. We used this to perform a merged Louvain clustering of the two datasets and UMAP representation (Supplementary Fig. 10).

To identify cell types, we used top differentially open regions (top 1600 most differential between all Louvain clusters) and associated them to their closest gene if they are in the ±10kb vicinity of it. This left a list of 1056 peaks associated to 984 genes. We crossed these genes to a list of known brain marker genes to determine cell types (Supplementary Fig. 10).

BENCHMARKING TO OTHER scATAC-seq METHODS

For the benchmarking of epiScanpy against 11 other scATAC-seq tools using four different real datasets, we used the framework from Chen et al.[15] provided at https://github.com/pinellolab/scATAC-benchmarking/ (datasets and Jupyter notebooks). The datasets used are the whole Cusanovich et al. scATAC-seq mouse Atlas[14] and the Buenrostro et al. scATAC-seq dataset[13] of human hematopoietic cells. The results of the benchmarking from Chen et al.[15] are compared to the ones that we obtained for epiScanpy, including runtime and memory usage, and are summarized in Supplementary Fig. 13.

For comparison in terms of runtime and memory usage, the best performing methods, cisTopic, was selected. The benchmark was performed using CentOS Linux 7 running on an AMD Opteron 6376 2.3GHz with 245/45.2 MB/s of input/output speeds. The number of concurrent CPU cores was set to 16, and the

maximum number of memory was set to 180GB. The actual memory usages and the run times of all runs were measured by Slurm[22]. We used cisTopic version 0.3.0, obtained from https://github.com/aertslab/cisTopic, running on R version 3.6.1. For epiScanpy, we used version 0.2.2 running on Python version 3.7.9 with scanpy version 1.6.0 and AnnData version 0.7.4. The benchmarked analysis parts of cisTopic and epiScanpy consisted of the following steps: reading input files, filtering features and cells for quality control, identifying groups of cells, and saving analysis results and plots. We benchmarked on 4 data scenarios, which are 1) Buenrostro et al. 2018 dataset from bulk peaks, 2) Buenrostro et al. 2018 dataset with 150,429 filtered features, 3) Cusanovich et al. mouse atlas downsampled dataset to 12,178 cells, and 4) full Cusanovich et al. mouse atlas with 81,173 cells. As shown in Supplementary Fig. 13, both epiScanpy and cisTopic are comparable in terms of runtime. For memory usage, epiScanpy consumed less memory (approximately half of the memory used by cisTopic). Because of the memory issue, cisTopic was unable to construct the data matrix for the full Cusanovich et al. dataset from the bam files using the function createcisTopicObjectFromBAM (cisTopic could not finish running and it was killed after 62.94 hours during data matrix construction, as it had reached the memory limit of 233 GB, using 180GB of RAM + 43GB of virtual memory). However, we transformed and inputted the data matrix built by epiScanpy to benchmark the performance of cisTopic for the analysis part. For that, we did input the count matrix generated by epiScanpy as a sparse matrix to cisTopic using the R package Seurat v3 (https://www.cell.com/cell/fulltext/S0092-8674(19)30559-8) and using the function createcisTopicObject. It took cisTopic 2 days 3 hours 22 minutes 23 seconds and 23.42GB of memory usage to build eight topic models (10, 20, 30, 40, 50, 60, 70 and 80 topics), and select the model with the best log-likelihood (80 topics; the log-likelihood had reached saturation).

CODE AVAILABILITY

All code used for the analysis of the data, as well as for the benchmarking, is available at https://github.com/colomemaria/episcanpy-paper/.

1. Pliner, H. A. *et al.* Cicero Predicts cis-Regulatory DNA Interactions from Single-Cell Chromatin Accessibility Data. *Mol. Cell* **71**, 858–871.e8 (2018).
2. Stuart, T. *et al.* Comprehensive Integration of Single-Cell Data. *Cell* (2019) doi:10.1016/j.cell.2019.05.031.
3. Blondel, V. D., Guillaume, J.-L., Lambiotte, R. & Lefebvre, E. Fast unfolding of communities in large networks. *J. Stat. Mech.* **2008**, P10008 (2008).
4. Haghverdi, L., Büttner, M., Wolf, F. A., Buettner, F. & Theis, F. J. Diffusion pseudotime robustly reconstructs lineage branching. *Nat. Methods* **13**, 845–848 (2016).

5. McInnes, L., Healy, J. & Melville, J. UMAP: Uniform Manifold Approximation and Projection for Dimension Reduction. (2018).
6. Maaten, L. van der & Hinton, G. Visualizing Data using t-SNE. *J. Mach. Learn. Res.* **9**, 2579–2605 (2008).
7. Wolf, F. A. *et al.* PAGA: graph abstraction reconciles clustering with trajectory inference through a topology preserving map of single cells. *Genome Biol.* **20**, 59 (2019).
8. Ntranos, V., Yi, L., Melsted, P. & Pachter, L. Identification of transcriptional signatures for cell types from single-cell RNA-Seq. *bioRxiv* 258566 (2018) doi:10.1101/258566.
9. Luo, C. *et al.* Single-cell methylomes identify neuronal subtypes and regulatory elements in mammalian cortex. *Science* **357**, 600–604 (2017).
10. Fang, R., Preissl, S., Hou, X., Lucero, J. & Wang, X. Fast and Accurate Clustering of Single Cell Epigenomes Reveals Cis-Regulatory Elements in Rare Cell Types. *bioRxiv* (2019).
11. Datasets - Single Cell ATAC - Official 10x Genomics Support. https://support.10xgenomics.com/single-cell-atac/datasets.
12. Satpathy, A. T. *et al.* Massively parallel single-cell chromatin landscapes of human immune cell development and intratumoral T cell exhaustion. *Nat. Biotechnol.* **37**, 925–936 (2019).
13. Buenrostro, J. D. *et al.* Integrated Single-Cell Analysis Maps the Continuous Regulatory Landscape of Human Hematopoietic Differentiation. *Cell* **173**, 1535–1548.e16 (2018).
14. Cusanovich, D. A. *et al.* A Single-Cell Atlas of In Vivo Mammalian Chromatin Accessibility. *Cell* **174**, 1309–1324.e18 (2018).
15. Chen, H. *et al.* Assessment of computational methods for the analysis of single-cell ATAC-seq data. *Genome Biol.* **20**, 241 (2019).
16. Dreos, R., Ambrosini, G., Groux, R., Cavin Périer, R. & Bucher, P. The eukaryotic promoter database in its 30th year: focus on non-vertebrate organisms. *Nucleic Acids Res.* **45**, D51–D55 (2017).
17. Shen, Y. *et al.* A map of the cis-regulatory sequences in the mouse genome. *Nature* **488**, 116–120 (2012).
18. Hinrichs, A. S. *et al.* The UCSC Genome Browser Database: update 2006. *Nucleic Acids Res.* **34**, D590–8 (2006).
19. Zhang, Y. *et al.* Model-based analysis of ChIP-Seq (MACS). *Genome Biol.* **9**, R137 (2008).
20. Pedregosa, F. *et al.* Scikit-learn: Machine Learning in Python. *J. Mach. Learn. Res.* **12**, 2825–2830 (2011).
21. Polański, K. *et al.* BBKNN: fast batch alignment of single cell transcriptomes. *Bioinformatics* **36**, 964–965 (2020).
22. Yoo, A. B., Jette, M. A. & Grondona, M. SLURM: Simple Linux Utility for Resource Management. in *Job Scheduling Strategies for Parallel Processing* 44–60 (Springer Berlin Heidelberg, 2003).

Supplementary figures

EpiScanpy: integrated single-cell epigenomic analysis

Authors: Anna Danese[1], Maria L. Richter[1], Kridsadakorn Chaichoompu[1], David S. Fischer[1,3], Fabian J. Theis[1,2,3]*, Maria Colomé-Tatché[1,2]*

Affiliations:
[1] Institute of Computational Biology, Helmholtz Center Munich, German Research Center for Environmental Health, Neuherberg, Germany
[2] TUM School of Life Sciences Weihenstephan, Technical University of Munich, Freising, Germany
[3] Department of Mathematics, Technical University of Munich, Garching, Germany

* correspondence to:
fabian.theis@helmholtz-muenchen.de and maria.colome@helmholtz-muenchen.de

Suppl. Figure 1: Quality control plots for scATAC-seq data

a)

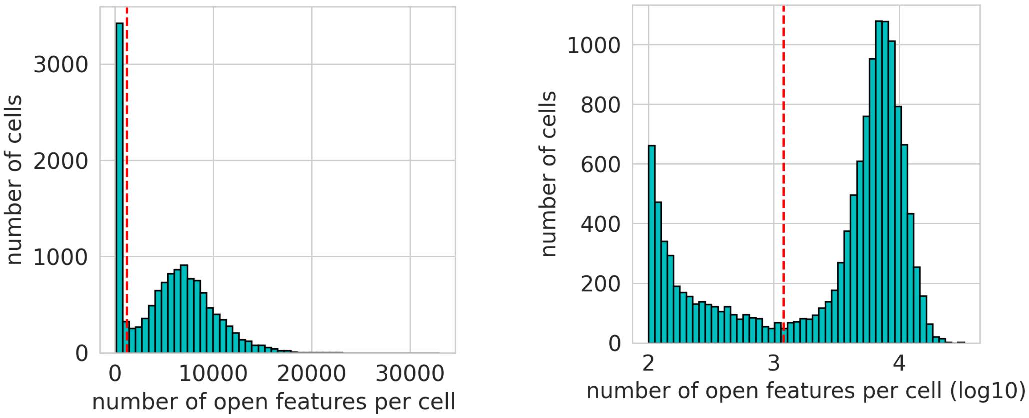

b)

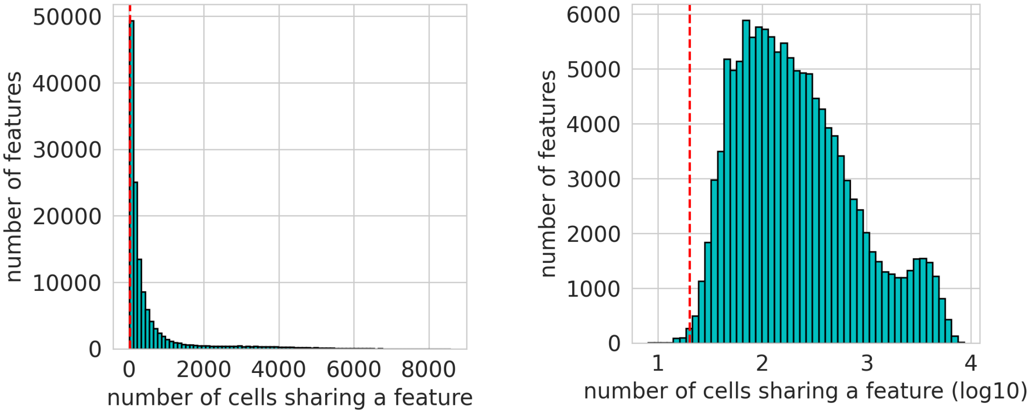

c)

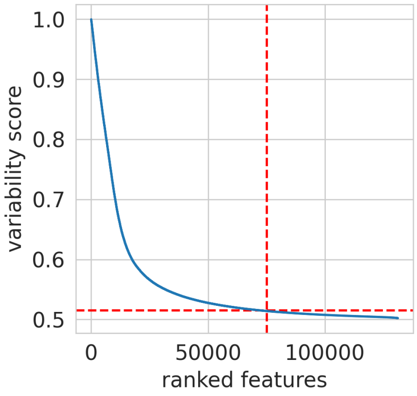

Suppl. Figure 1 - 1: Quality control plots for scATAC-seq data: 10x PBMC data (13,557 cells x 131,562 peaks): a) Histogram of cell coverage (scATAC-seq) between all cells. **b**) Histogram of feature coverage for peaks (scATAC-seq). **c**) Variability score per feature with ranked features. The red lines represent the cut-off. In the histrograms **a** and **b**, the values on the right of the vertical red lines are kept. In the plot **c**, the values above the horizontal red line and the values on the left of the vertical red line are kept.

Suppl. Figure 1: Quality control plots for scATAC-seq data

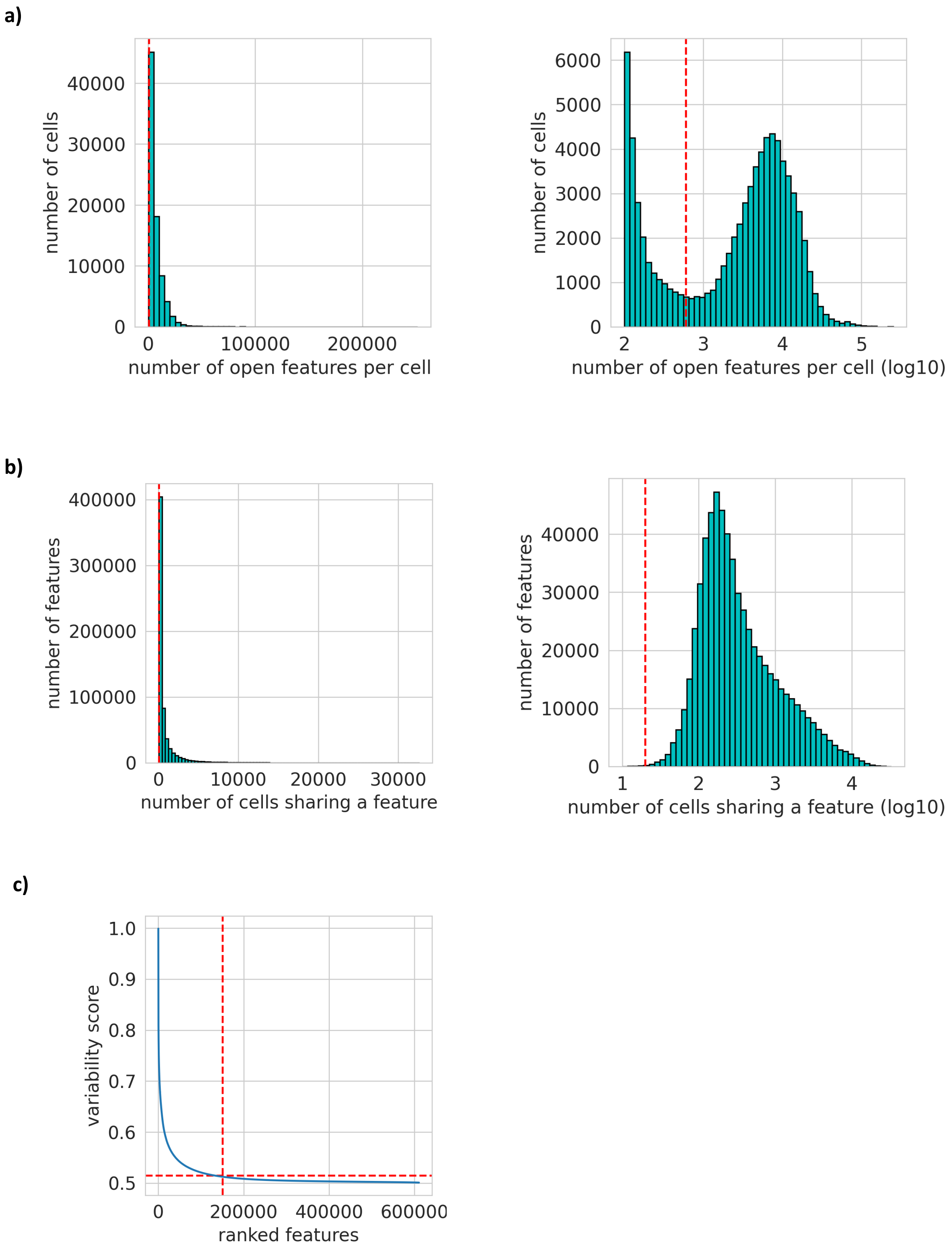

Suppl. Figure 1 - 2: Quality control plots for scATAC-seq data: Satpathy et al 2019 data (79,370 cells x 618,737 peaks): a) Histogram of cell coverage (scATAC-seq) between all cells. **b**) Histogram of feature coverage for peaks (scATAC-seq). **c**) Variability score per feature with ranked features. The red lines represent the cut-off. In the histrograms **a** and **b**, the values on the right of the vertical red lines are kept. In the plot **c**, the values above the horizontal red line and the values on the left of the vertical red line are kept.

Suppl. Figure 2: Quality control plots for single-cell DNA methylation data

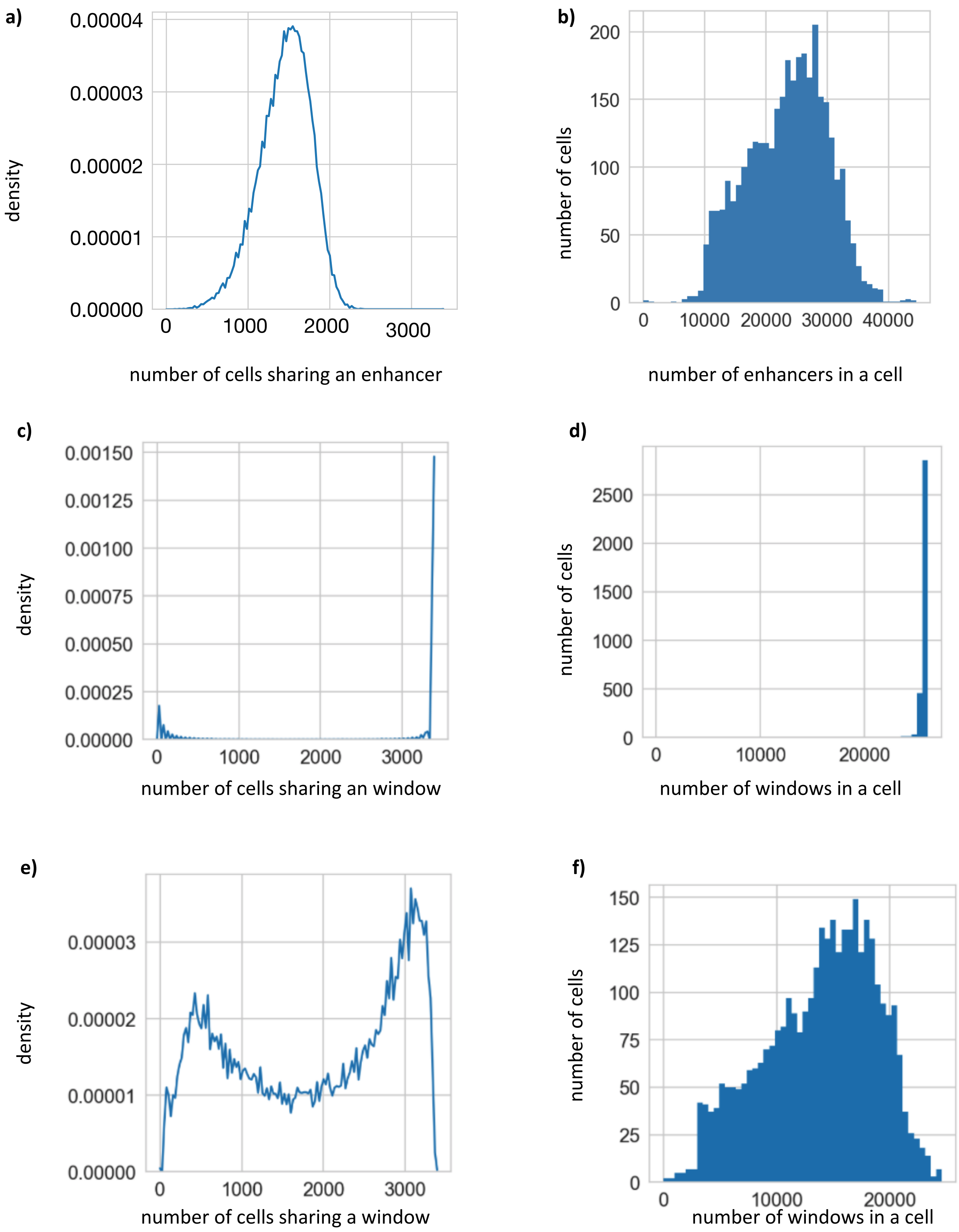

Suppl. Figure 2 (continued)

g)

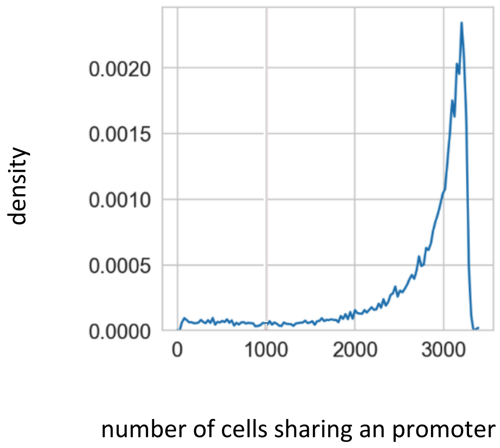

h)

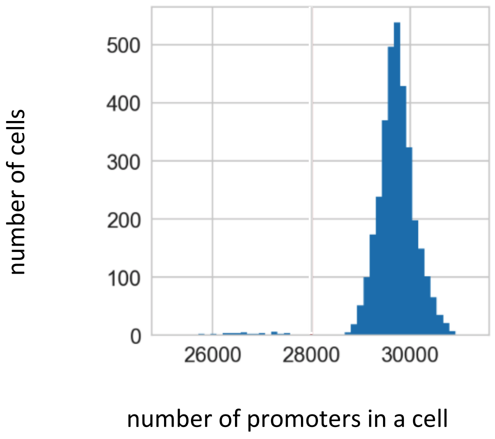

Suppl. Figure 2: Quality control plots for single-cell DNA methylation data: a) Density distribution of enhancer commonness (DNA methylation) between all cells, for enhancers with at least 1 covered cytosine in CG context (3,388 cells x 55019 enhancers). **b)** Histogram of the number of enhancers per cell, with at least 1 CG covered**. c)** Density distribution of 100 kb window commonness (DNA methylation) between all cells, for windows with at least 1 covered cytosine in CG context. **d)** Histogram of the number of windows per cell, with at least 1 CG covered (3,388 cells x 41,980 windows). **e), f)** Same as c) and d) but with windows with at least 30 covered CGs (3,388 cells x 41,980 windows). **g)** Density distribution of promoter commonness (DNA methylation) between all cells, for promoters with at least 1 covered cytosine in CG context (3,388 cells x 37,546 promoters). **h)** Histogram of the number of promoters per cell, with at least 1 CG covered.

Suppl. Figure 3: Extra quality control plots for scATAC-seq data

a)

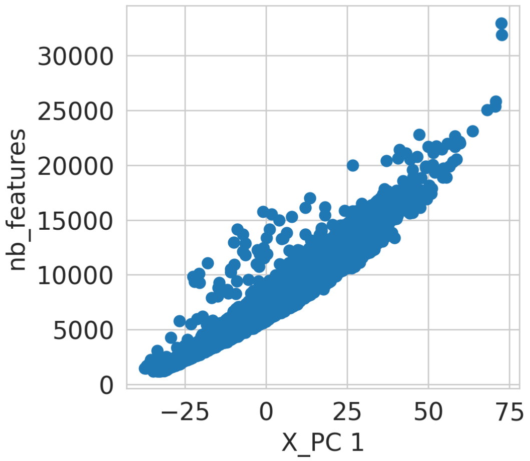

b)

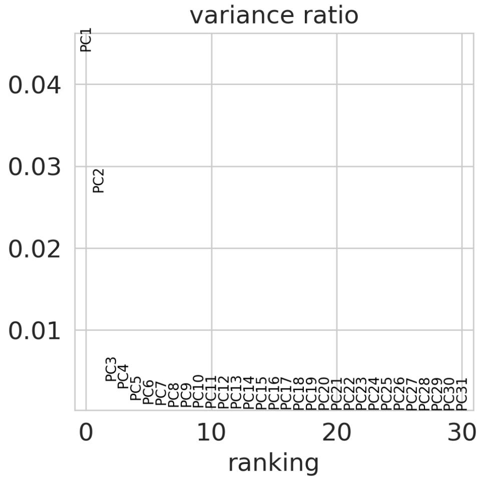

Suppl. Figure 3: Extra quality control plots for scATAC-seq data. a) PC1 is correlated with cell coverage (data from the 10x PBMCs, 9,891 cells x 75,226 peaks). **b)** variance ratio per principal component (data from the 10x PBMCs).

Suppl. Figure 4: Comparison of single-cell DNA methylation data clustering between feature spaces

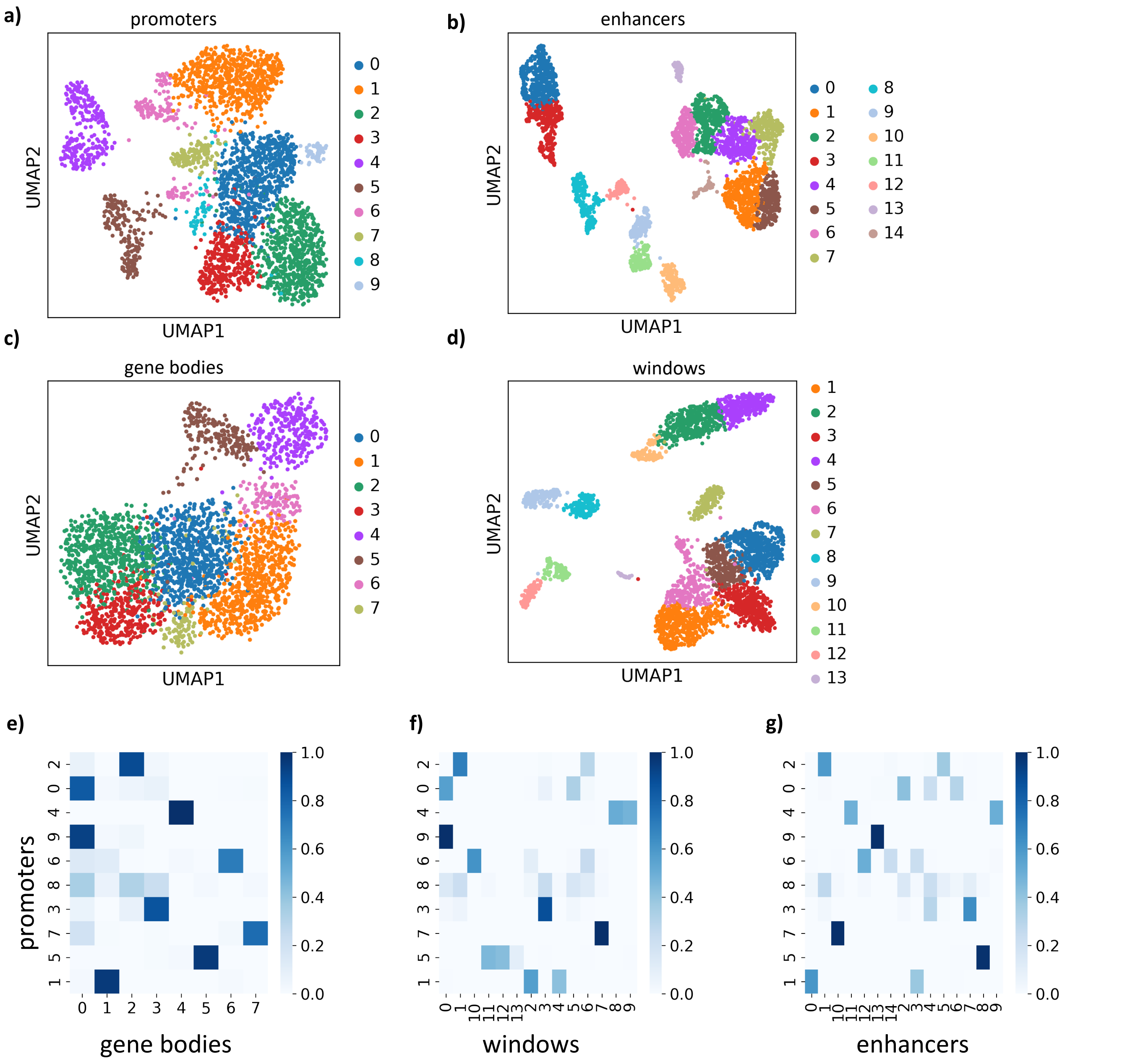

Suppl. Figure 4: Comparison of single-cell DNA methylation data clustering between feature spaces: sc DNA methylation clustering for prefrontal cortex neurons using different feature spaces, using Louvain clustering with resolution = 1: **a)** promoters CG methylation (3,224 cells x 32,610 promoters), **b)** enhancer CG methylation (3,288 cells x 54,932 enhancers), **c)** gene body CG methylation (3,019 cells x 26,947 gene bodies), **d)** 100kb windows (3,223 cells x 6,729 windows). **e-g)** heatmap for cluster identification comparison between **e)** promoters and gene bodies (3,018 cells), **f)** promoter and windows (3,222 cells) and **g)** promoters and enhancers (3,193 cells). In all cases, Louvain clustering was done with resolution = 1.

Suppl. Figure 5: Silhouette score for different single-cell DNA methylation data clustering between feature spaces

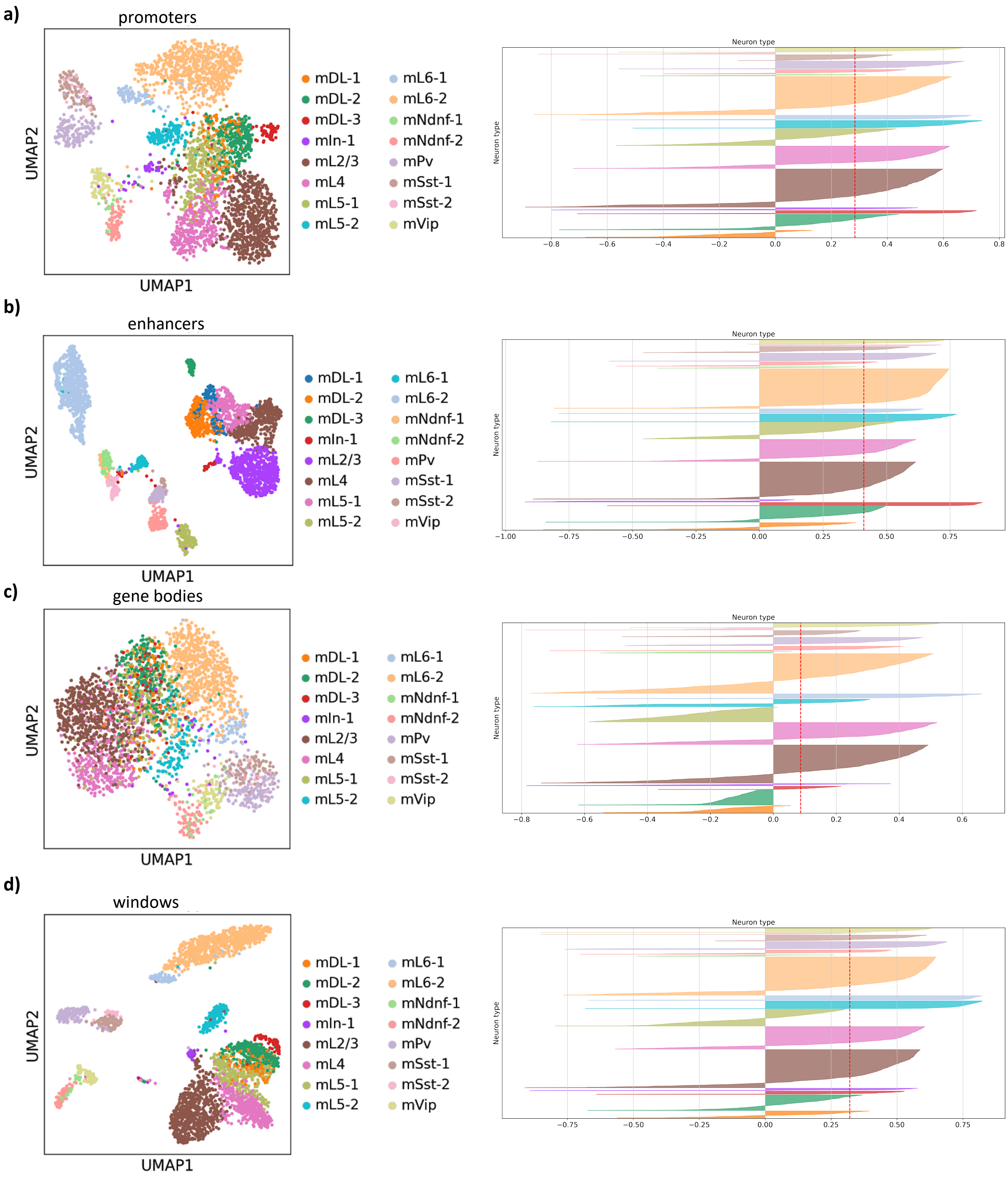

Suppl. Figure 5: Silhouette score for different single-cell DNA methylation data clustering between feature spaces: Left: UMAP embedding in different feature spaces (3,224 cells x 32,610 promoters **(a)**, 3,288 cells x 54,932 enhancers **(b)**, 3,019 cells x 26,947 gene bodies **(c)** and 3,223 cells x 6,729 windows **(d)**) with cell type annotation (from the original paper, Luo et al 2017). Right: silhouette score in the corresponding feature space. The red vertical line represents the average silhouette score from all cells.

Suppl. Figure 6: Example of cell type identification using CG DNA methylation levels at the promoter of known neuronal markers

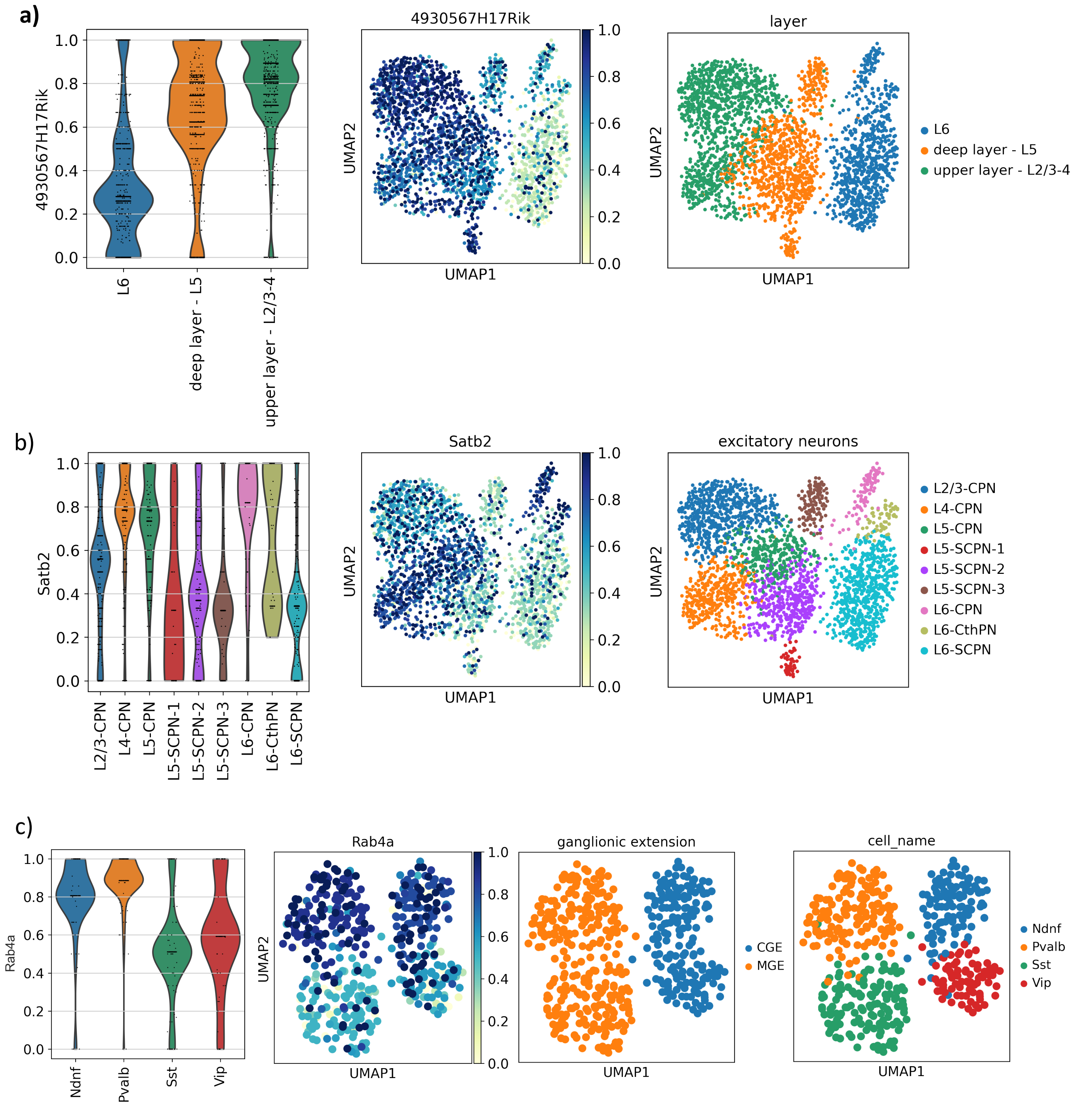

Suppl. Figure 6: Example of cell type identification using CG DNA methylation levels at the promoter of known neuronal markers: a) DNA methylation levels at *4930567H1Rik* (2,734 cells x 32,610 promoters). **b)** DNA methylation levels at *Satb2* (2,734 cells x 32,610 promoters). **c)** DNA methylation levels at *Rab4a* (449 cells x 32,610 promoters).

Suppl. Figure 7: Example of cell type identification using CH DNA methylation levels at the gene body of known neuronal markers

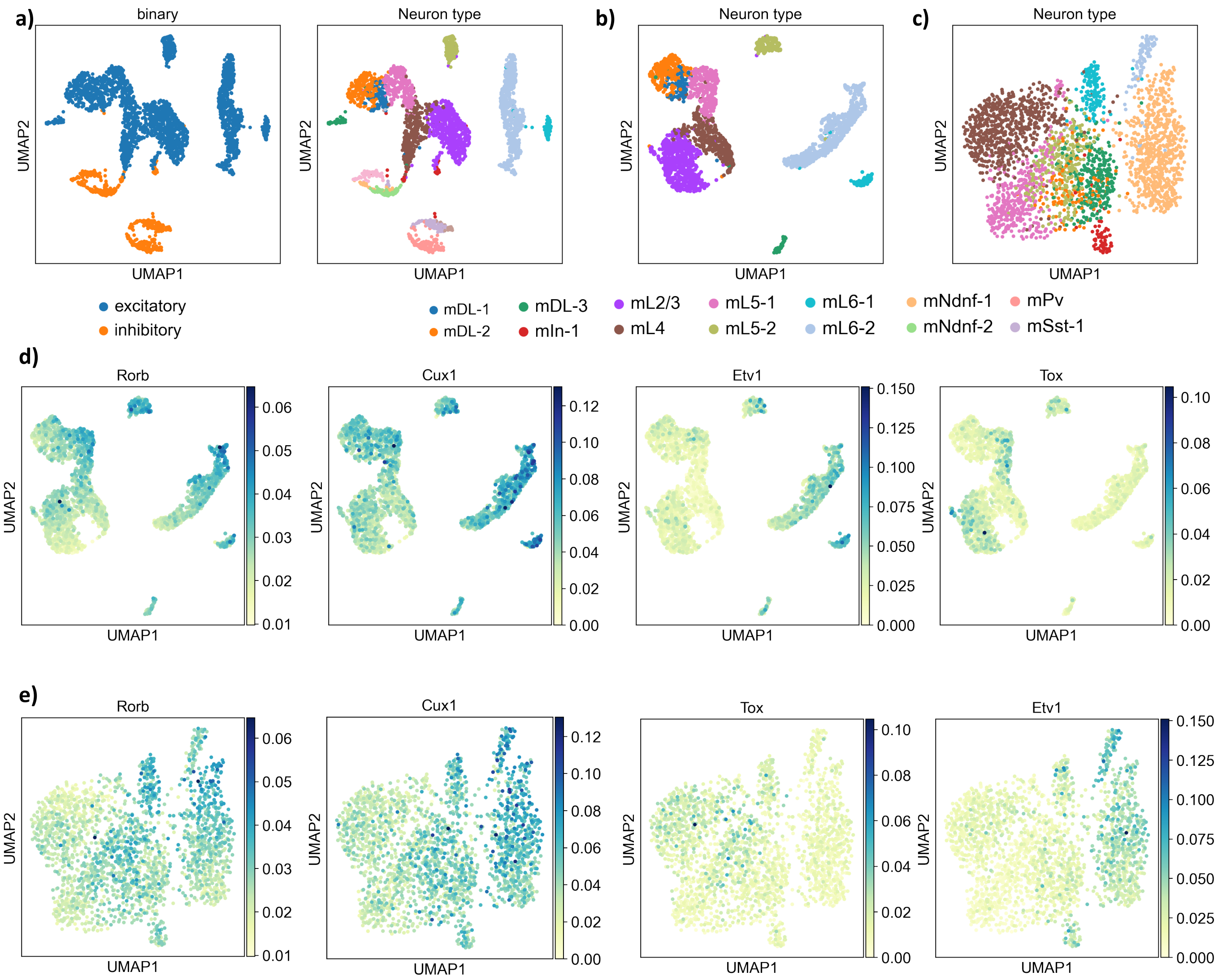

Suppl. Figure 7: Example of cell type identification using CH DNA methylation levels at the promoter of known neuronal markers: a) UMAP with CH gene body embedding with cell labels for excitatory and inhibitory neurons (2,876 cells x 25,489 gene bodies) **b,c)** Excitatory neurons labelled per Neuron type, with b) UMAP with CH gene body embedding (2,423 cells x 25,489 gene bodies) and c) UMAP with CG promoter embedding(2,734 cells x 32,610 promoters) **d,e)** CH methylation levels at gene body of CPN (Rorb and Cux1) and SCPN (Etv1 and Tox) marker genes; with d**)** UMAP with CH gene body embedding e) UMAP with CG promoter embedding

Suppl. Figure 8: scATAC-seq hematopoiesis, top differential peaks linked to genes, 10x dataset

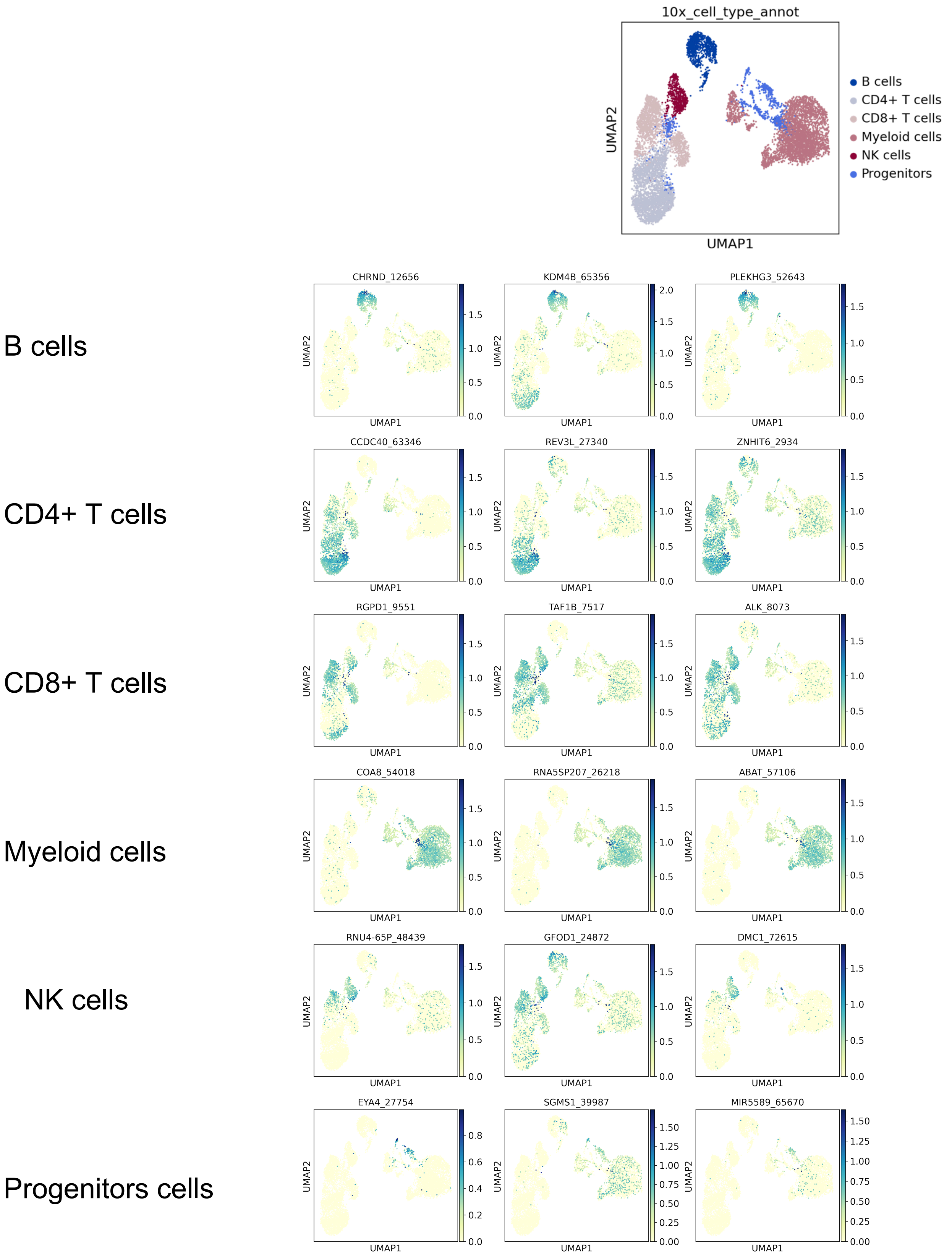

Suppl. Figure 8: scATAC-seq hematopoiesis, top differential peaks linked to genes: for the 10x dataset, openness at several marker genes per cell type (9,891 cells x 75,226 peaks).

Suppl. Figure 9: scATAC-seq hematopoiesis, Satpathy et al 2019

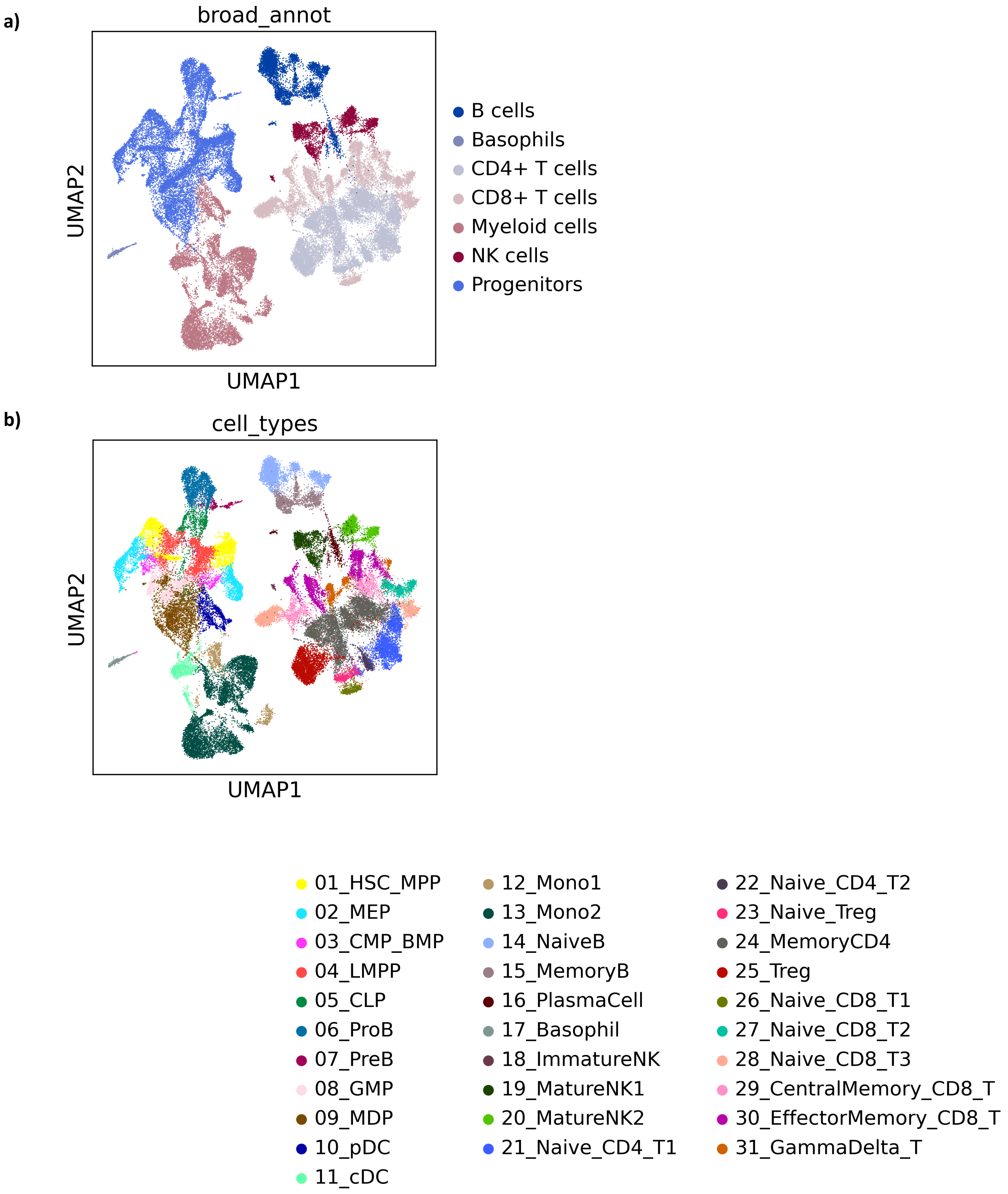

Suppl. Figure 9: scATAC-seq hematopoiesis, Satpathy et al 2019: embedding of the Satpathy et al. 2019 dataset with **(a)** broad cell type annotation and with **(b)** detailed cell type annotation (57,177 cells x 83,823 peaks)

Suppl. Figure 9: scATAC-seq hematopoiesis, Satpathy et al 2019, cell types

c)

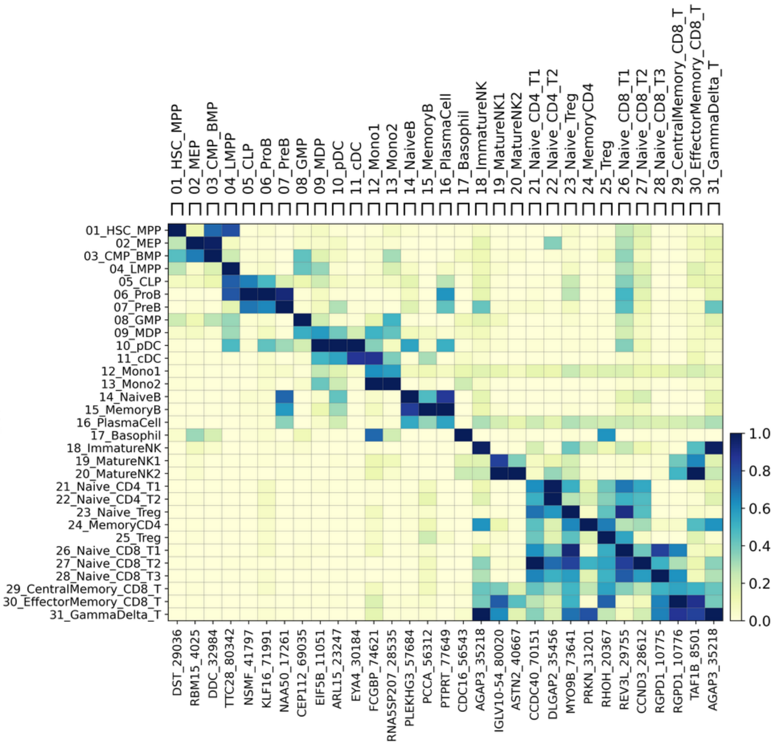

d)

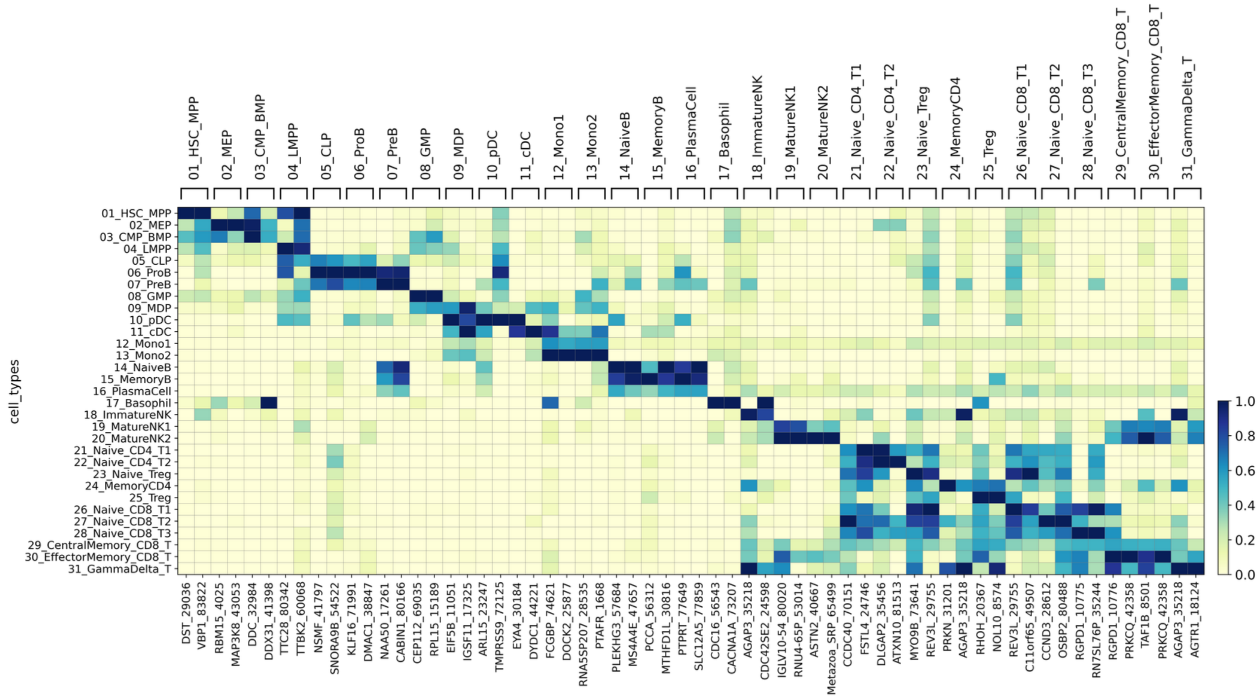

Suppl. Figure 9: scATAC-seq hematopoiesis, Satpathy et al 2019: Example of cell type identification plots: (57,177 cells x 83,823 peaks) **c)** Matrixplots showing, per annotated cell type, one of the top ranked open features that is within 5000bp upstream of a gene (gene names and peak index number are shown). **d)** Matrixplots showing, per annotated cell type, two of the top ranked open features that are within 5000bp upstream of a gene (gene names and peak index number are shown).

Suppl. Figure 9: scATAC-seq hematopoiesis, Satpathy et al 2019, cell types

e)

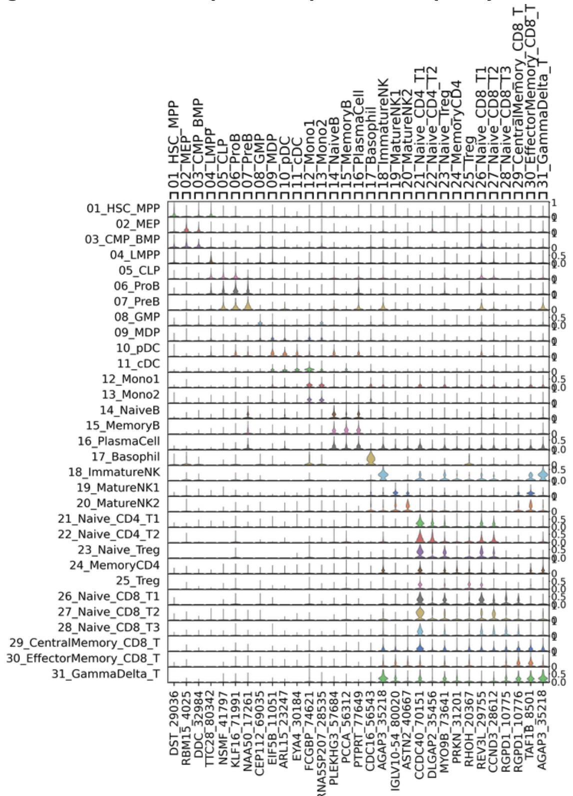

f)

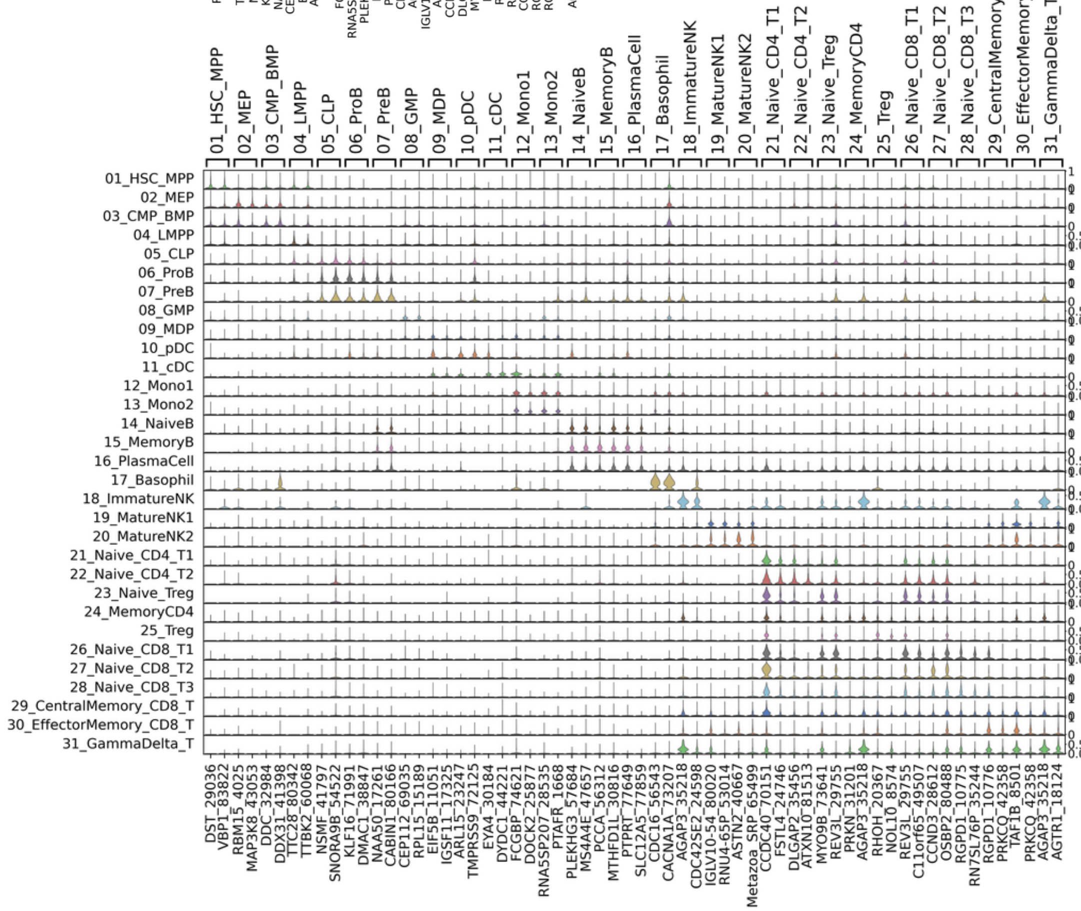

Suppl. Figure 9: scATAC-seq hematopoiesis, Satpathy et al 2019, Example of cell type identification plots : e) Stacked violin plot showing, per annotated cell type, one of the top ranked open features that is within 5000bp upstream of a gene (gene names and peak index number are shown). **f)** Stacked violin plot showing, per annotated cell type, two of the top ranked open features that are within 5000bp upstream of a gene (gene names and peak index number are shown).

Suppl. Figure 9: scATAC-seq hematopoiesis, Satpathy et al 2019, cell types

g)

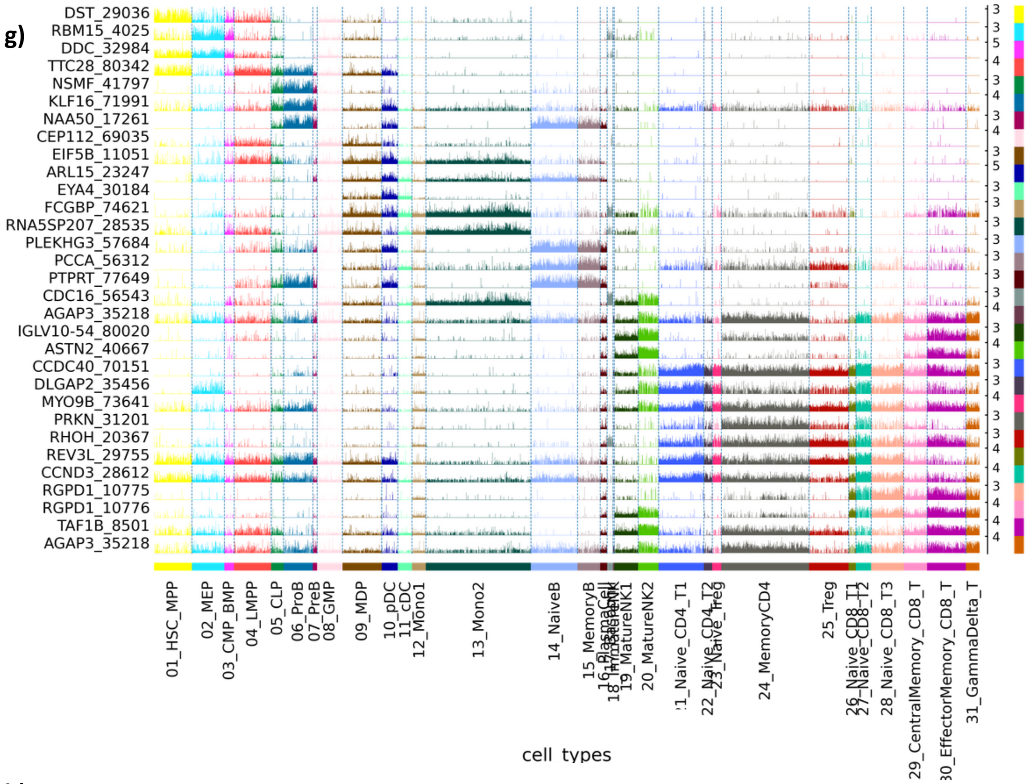

h)

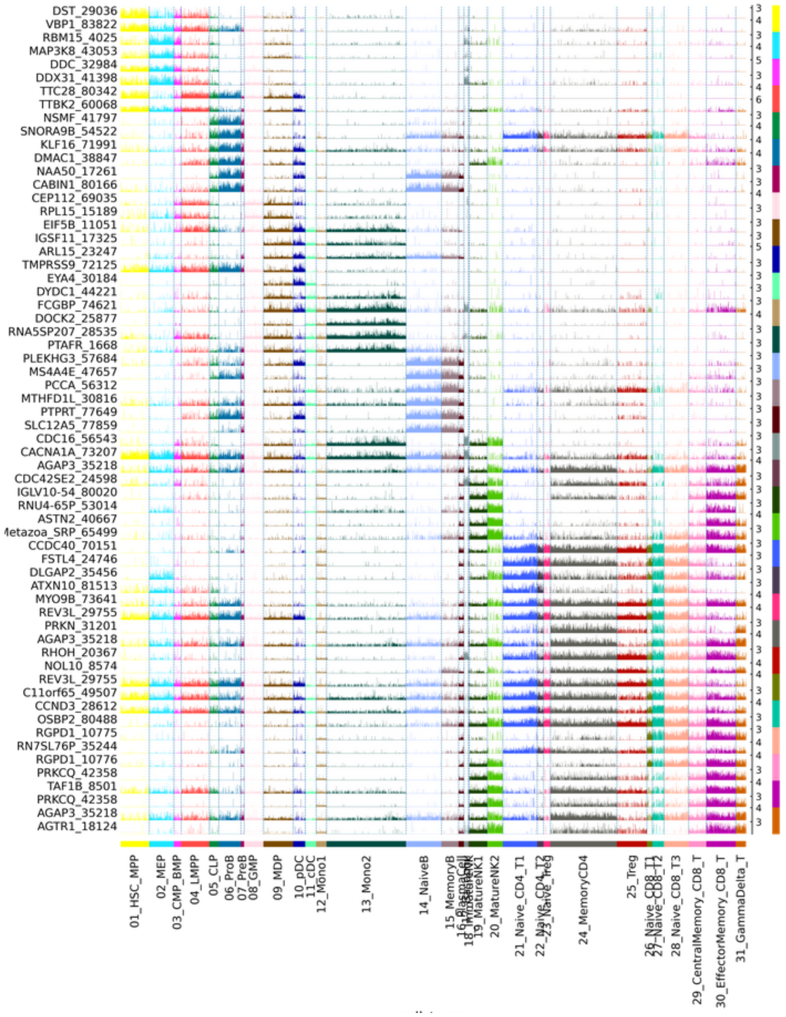

Suppl. Figure 9: scATAC-seq hematopoiesis, Satpathy et al 2019, Example of cell type identification plots : **g)** Tracks plot showing per annotated cell type one of the top ranked open features that is within 5000bp upstream of a gene (gene names and peak index number are shown). **h)** Tracks plot showing per annotated cell type two of the top ranked open features that are within 5000bp upstream of a gene (gene names and peak index number are shown).

Suppl. Figure 10: scATAC-seq dataset integration for mouse brain

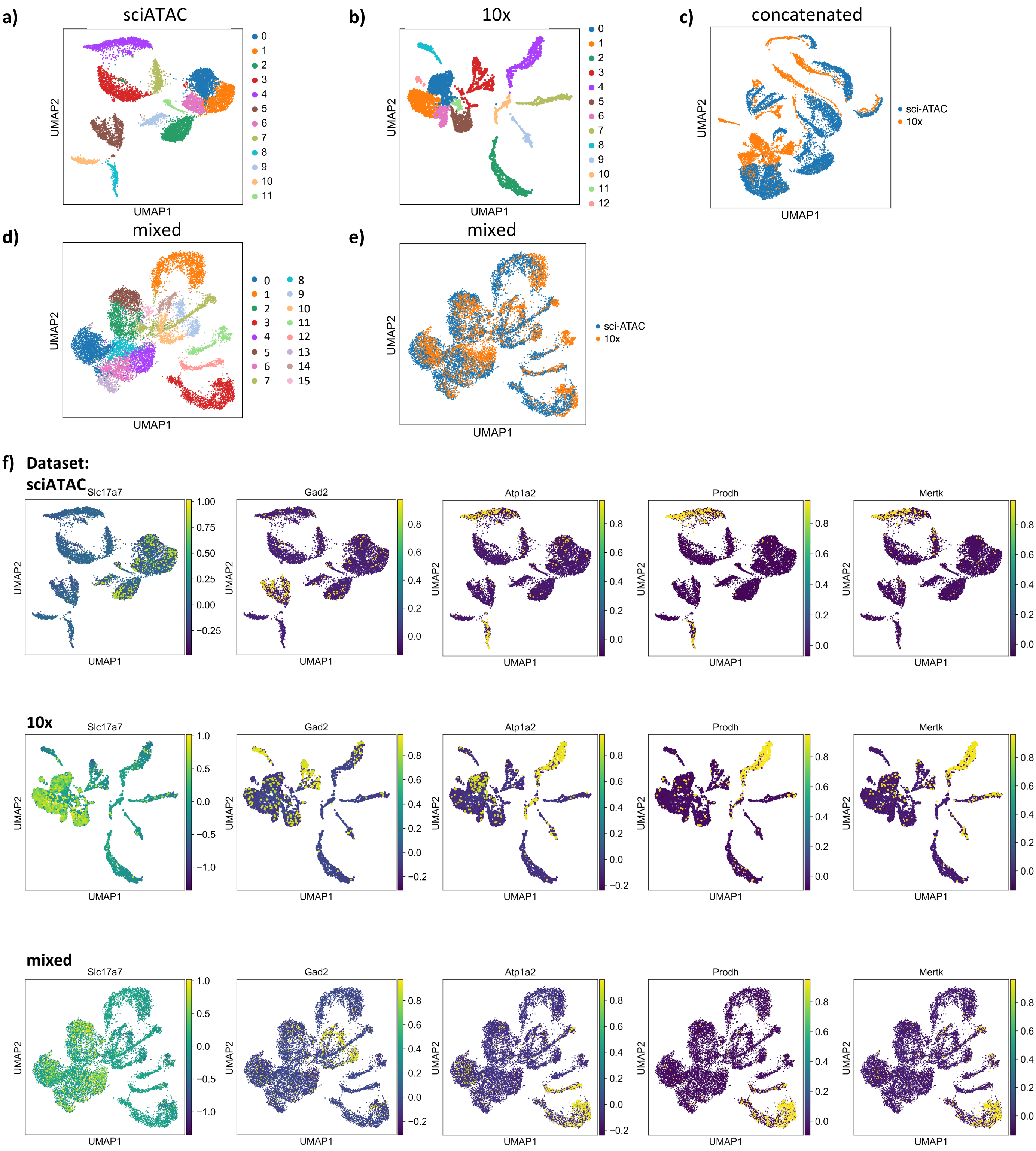

Suppl. Figure 10: scATAC-seq dataset integration, identification of cell type markers: a) UMAP for the brain sciATAC-seq data with Louvain clusters (9,145 cells x 54,539 windows); **b)** UMAP for the brain 10x data with Louvain cluster (4,044 cells x 54,539 windows); **c)** UMAP for the concatenated datasets with batch id (13,189 cells x 54,539 windows); **d)** UMAP for the concatenated and mixed (bb-knn) datasets with Louvain clusters; **e)** UMAP for the concatenated and mixed (bb-knn) datasets with batch id; **f)** example of openness at regions in a few marker genes that determine cell type (Slc17a7 is a marker of excitatory neurons and Gad2 for inhibitory neurons; Atp1a2, Prodh and Mertk are canonical markers for astrocytes).

Suppl. Figure 11: scATAC-seq hematopoiesis, Satpathy et al 2019, diffusion map:

a)

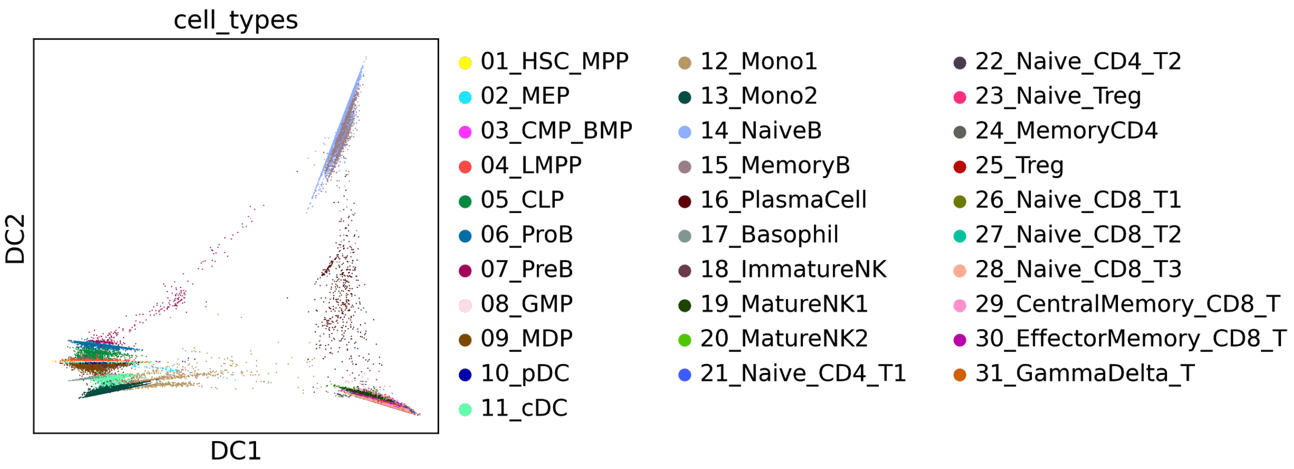

b)

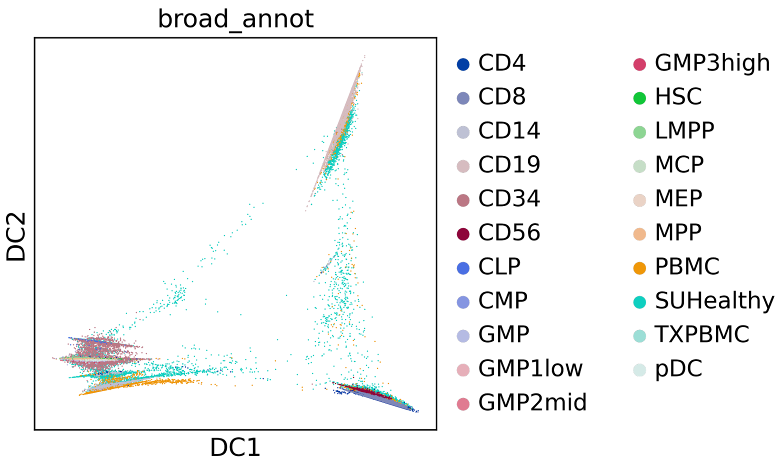

Suppl. Figure 11: scATAC-seq hematopoiesis, Satpathy et al 2019, diffusion map: 2D diffusion map with **a)** cell type annotation and **b)** broad cell type annotation (57,177 cells x 83,823 peaks).

Suppl. Figure 11: scATAC-seq hematopoiesis, Satpathy et al 2019, diffusion map:

c)

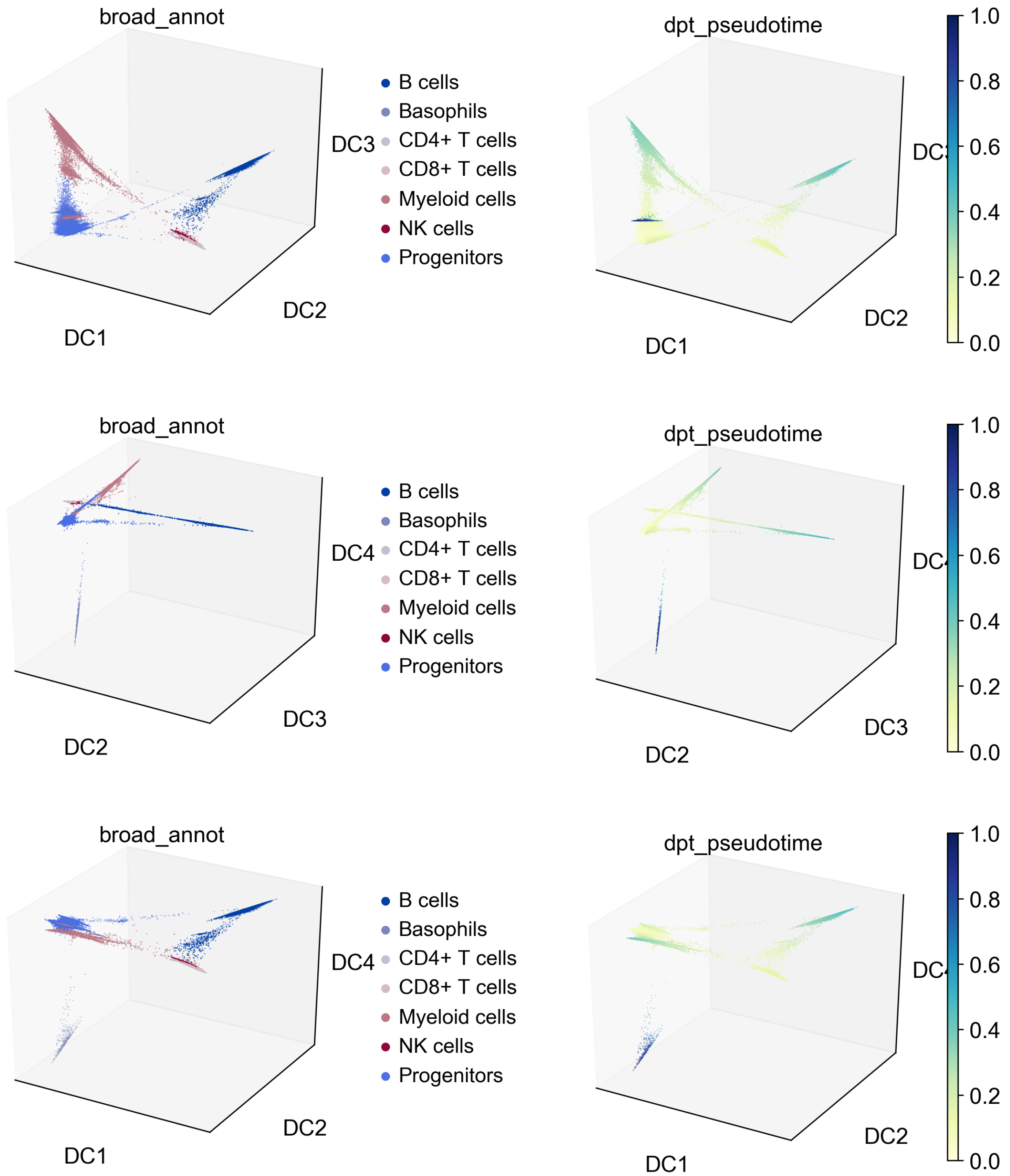

Suppl. Figure 11: scATAC-seq hematopoiesis, Satpathy et al 2019, diffusion map: c) 3D diffusion map with broad cell type annotation (left) and diffusion pseudotime (right).

Suppl. Figure 11: scATAC-seq hematopoiesis, Satpathy et al 2019, diffusion map:

d)

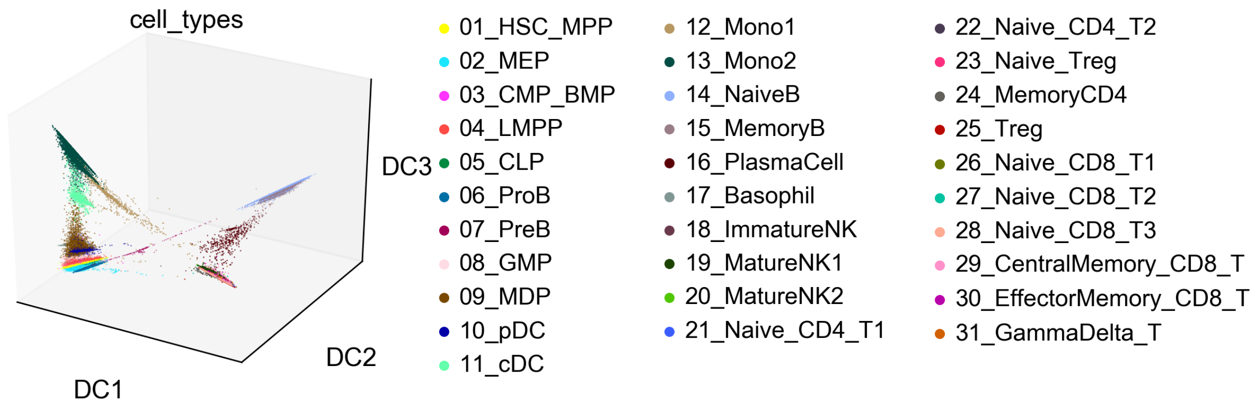

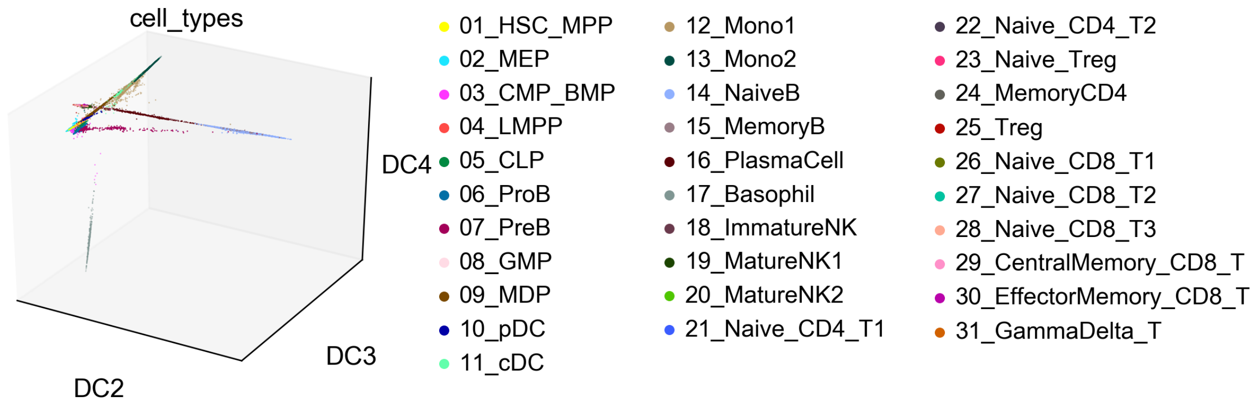

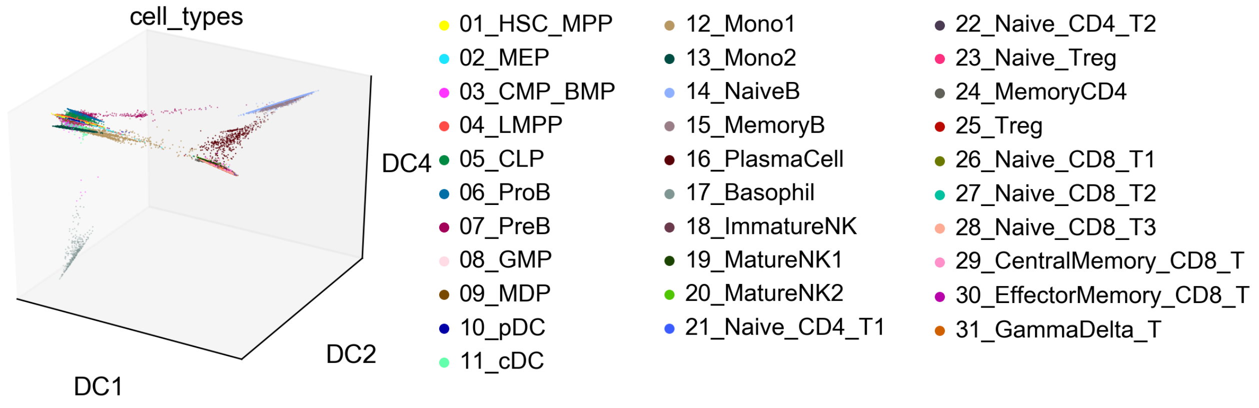

Suppl. Figure 11: scATAC-seq hematopoiesis, Satpathy et al 2019, diffusion map: d) 3D diffusion map with cell type annotation.

Suppl. Figure 12: scATAC-seq hematopoiesis, Satpathy et al 2019, differentiation paths:

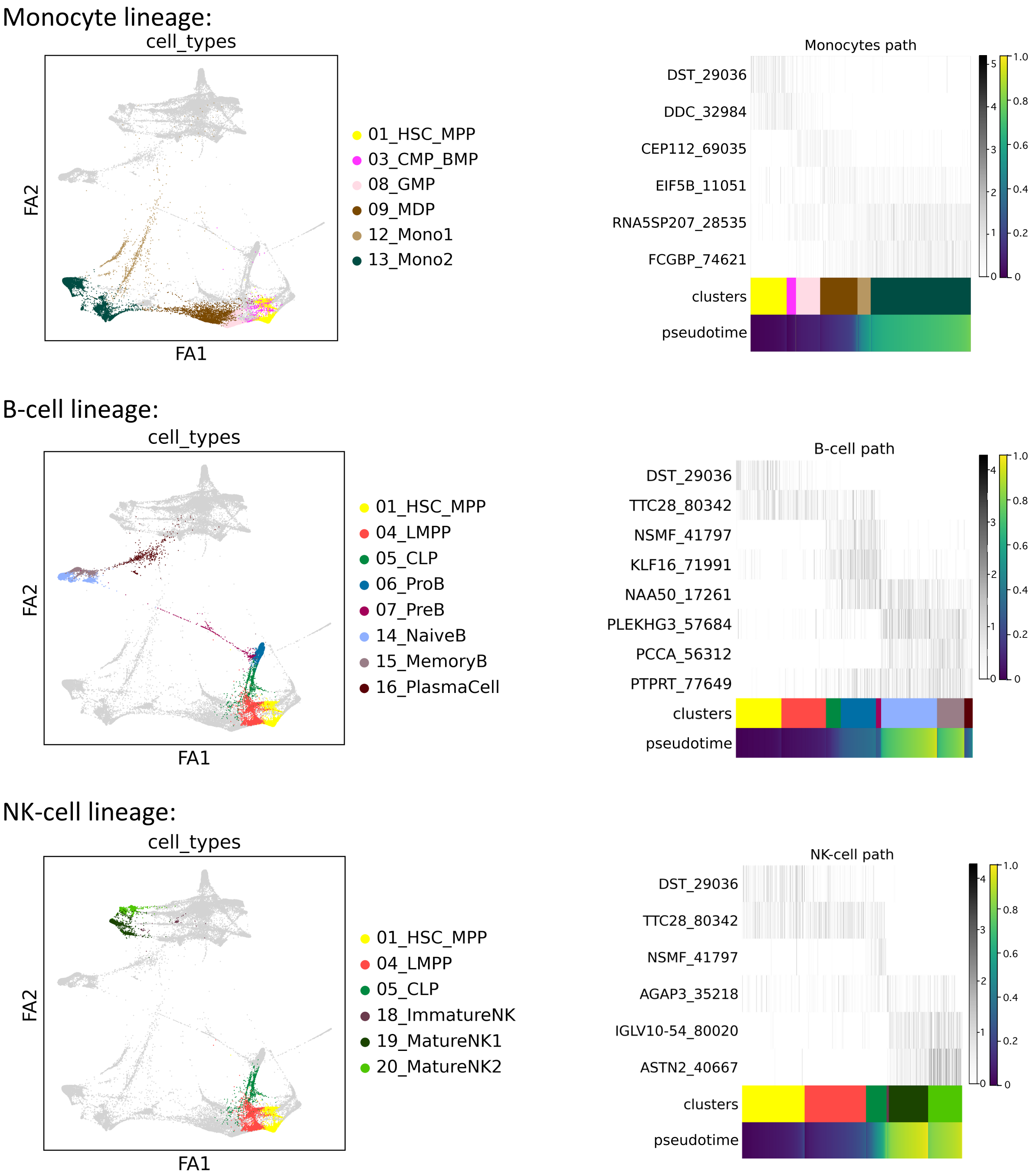

Suppl. Figure 12: scATAC-seq hematopoiesis, Satpathy et al 2019, differentiation paths: Force-directed graph drawing of the Satpathy et al. 2019 dataset, with different differentiation trajectories coloured (left) and the progressive opening and closing of marker peaks (gene name and peak index number) (right). The processed dataset contains 57,177 cells and 83,823 peaks. The monocyte lineage contains 16,004 cells, the B-cell lineage contains 13,656 cells, the NK-cell lineage contains 9,214 cells, the CD4+ T-cell lineage contains 15,838 cells, the CD8+ T-cell lineage contains 14,168 cells and the macrophage lineage contains 9,867 cells. The grey colour scale denotes the feature openness per cell, and the blue colour scale shows the pseudotime progression.

Suppl. Figure 12: scATAC-seq hematopoiesis

CD4+ T-cell lineage:

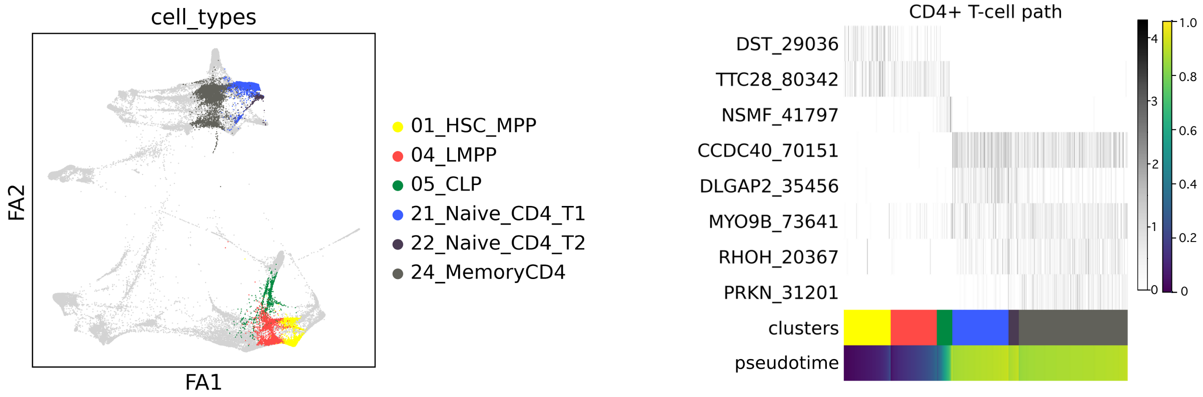

CD8+ T-cell lineage:

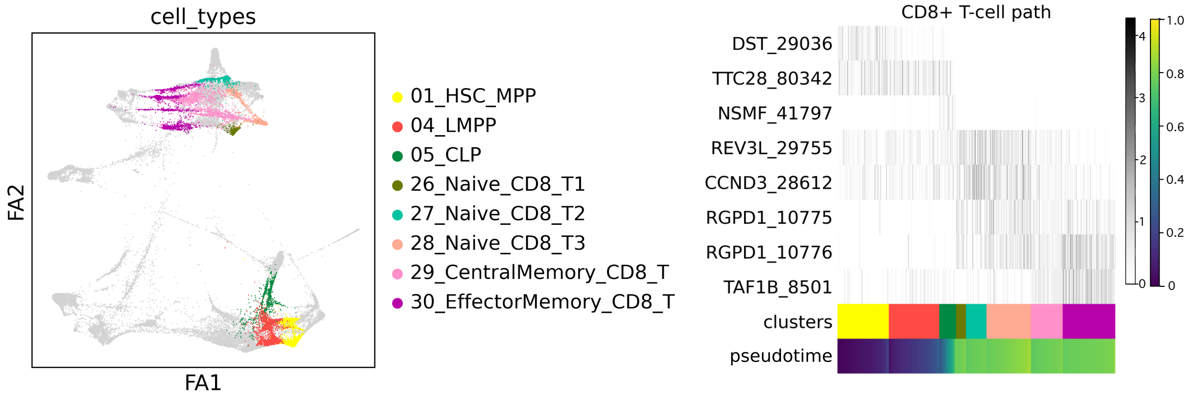

Macrophage lineage:

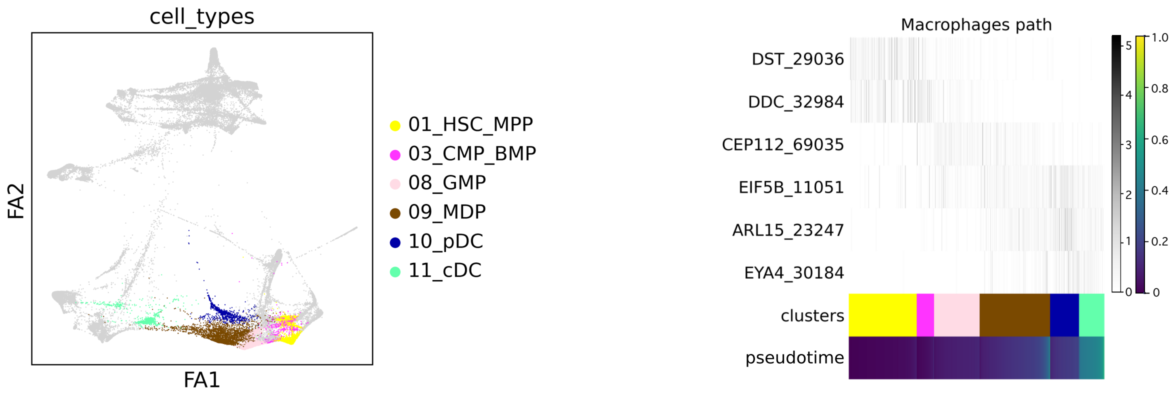

Suppl. Figure 12: scATAC-seq hematopoiesis, Satpathy et al 2019, differentiation paths: Force-directed graph drawing of the Satpathy et al. 2019 dataset, with different differentiation trajectories coloured (left) and the progressive opening and closing of marker peaks (gene name and peak index number) (right). The processed dataset contains 57,177 cells and 83,823 peaks. The monocyte lineage contains 16,004 cells, the B-cell lineage contains 13,656 cells, the NK-cell lineage contains 9,214 cells, the CD4+ T-cell lineage contains 15,838 cells, the CD8+ T-cell lineage contains 14,168 cells and the macrophage lineage contains 9,867 cells. The grey colour scale denotes the feature openness per cell, and the blue colour scale shows the pseudotime progression.

Suppl. Figure 13a: Benchmarking: epiScanpy on Buenrostro et al. dataset (bulk peaks)

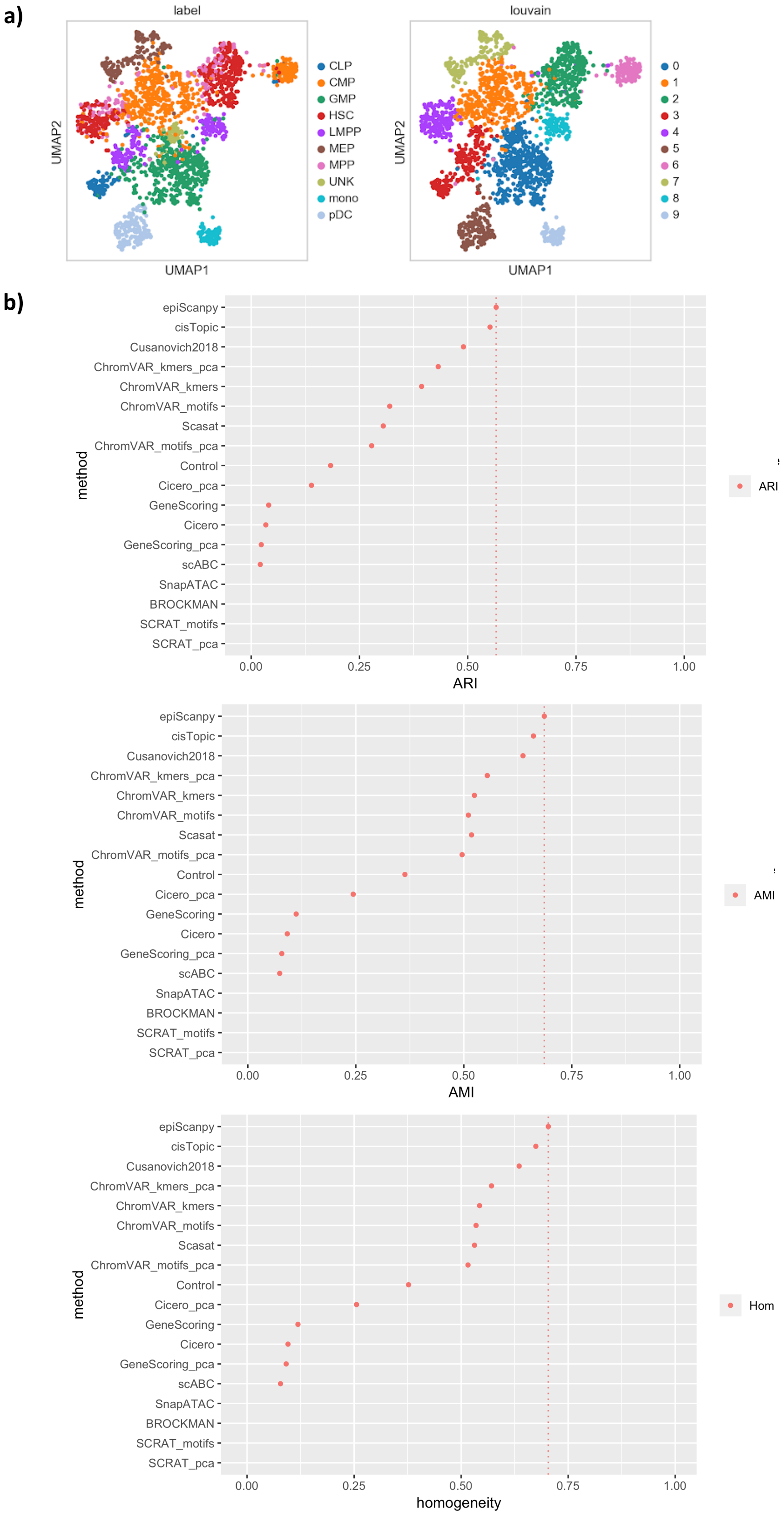

Suppl. Figure 13a: EpiScanpy performance on Buenrostro et al 2018 dataset, 2034 cells, using the bulk peaks feature space: a) UMAP with cell label and clustering results; **b)** metrics (ARI, AMI and Homogeneity).

Suppl. Figure 13b: Benchmarking: epiScanpy on Buenrostro et al. dataset

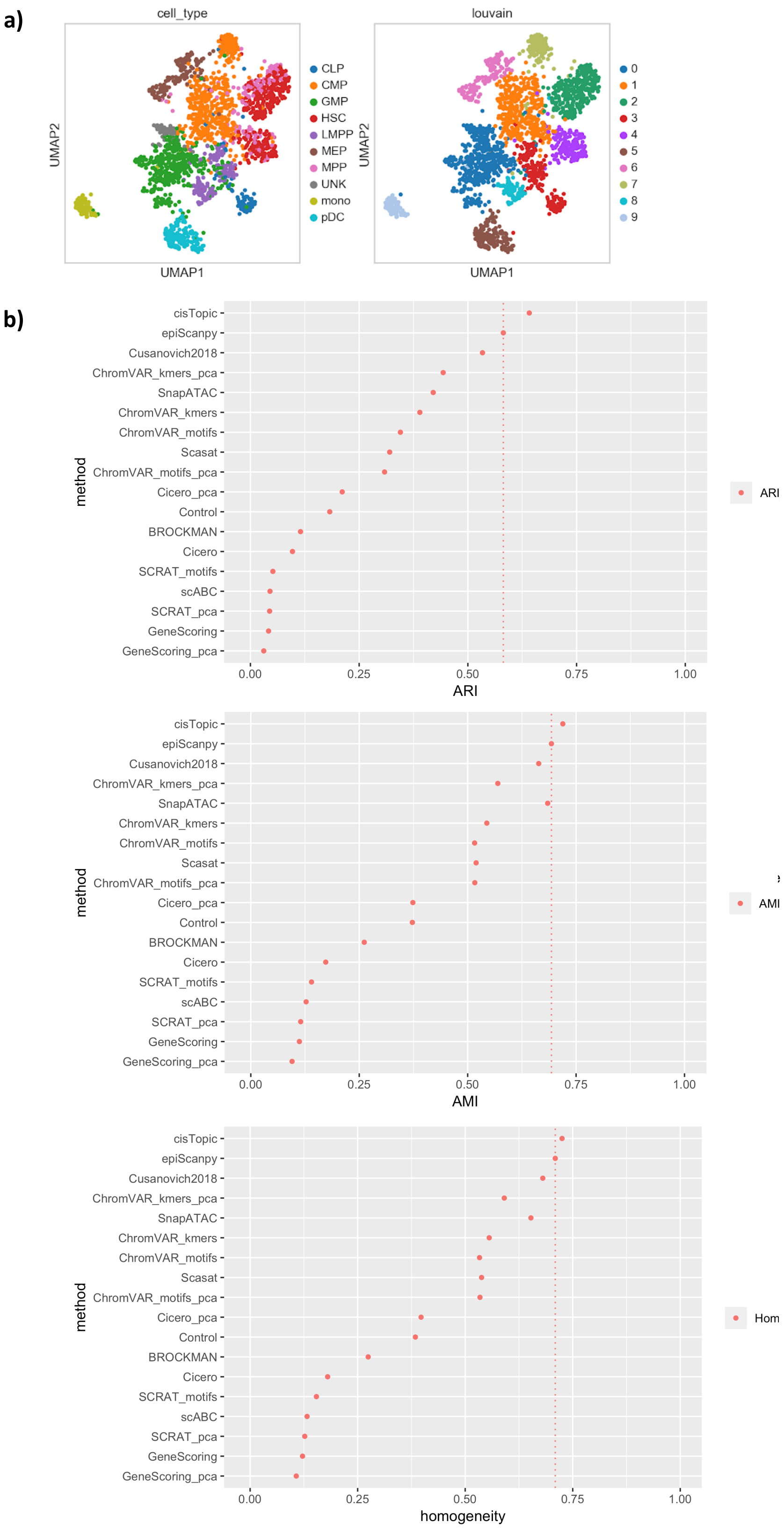

Suppl. Figure 13b: EpiScanpy performance on Buenrostro et al 2018 dataset, 2034 cells: a) UMAP with cell label and clustering results; **b)** metrics.

Suppl. Figure 13c: Benchmarking: epiScanpy on Cusanovich et al (subsampled)

a)

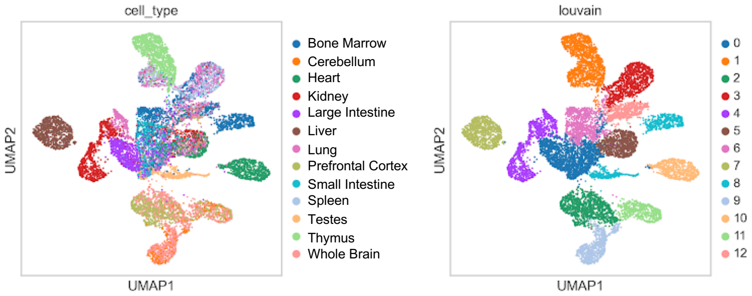

b)

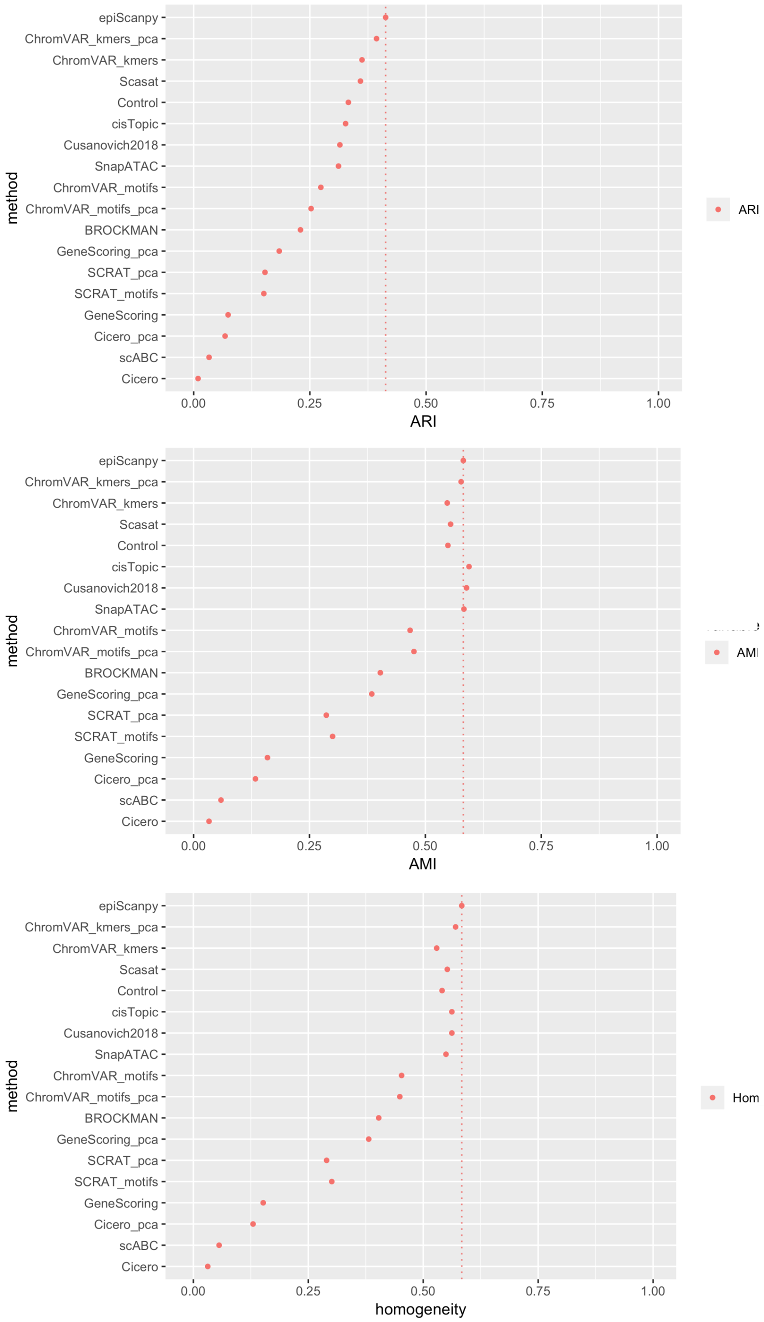

Suppl. Figure 13c: EpiScanpy performance using the downsampled Cusanovich et al. dataset with 12178 cells: a) UMAP with cell label and clustering results; **b)** metrics.

Suppl. Figure 13d: Benchmarking: epiScanpy on Cusanovich et al

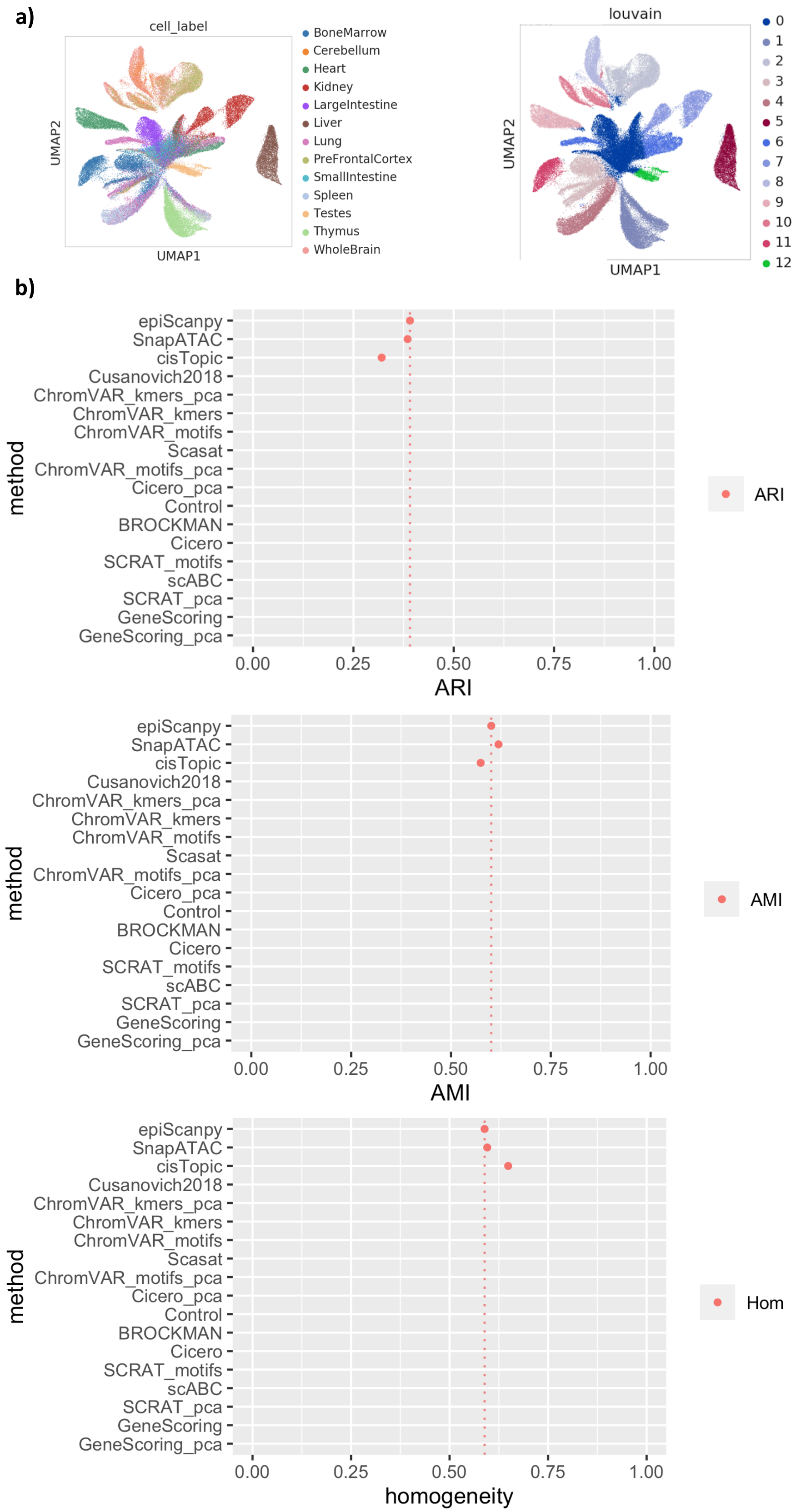

Suppl. Figure 13d: EpiScanpy performance using the full Cusanovich et al. dataset with 81173 cells: a) UMAP with cell label and clustering results; **b)** metrics.

Suppl. Figure 13e: Benchmarking: epiScanpy

a)

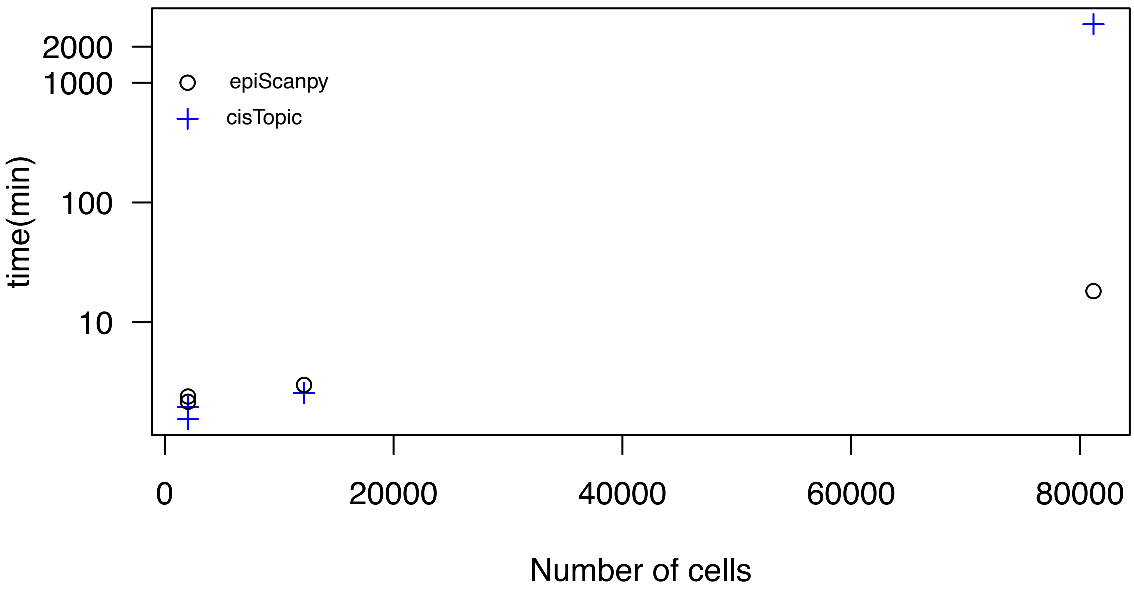

b)

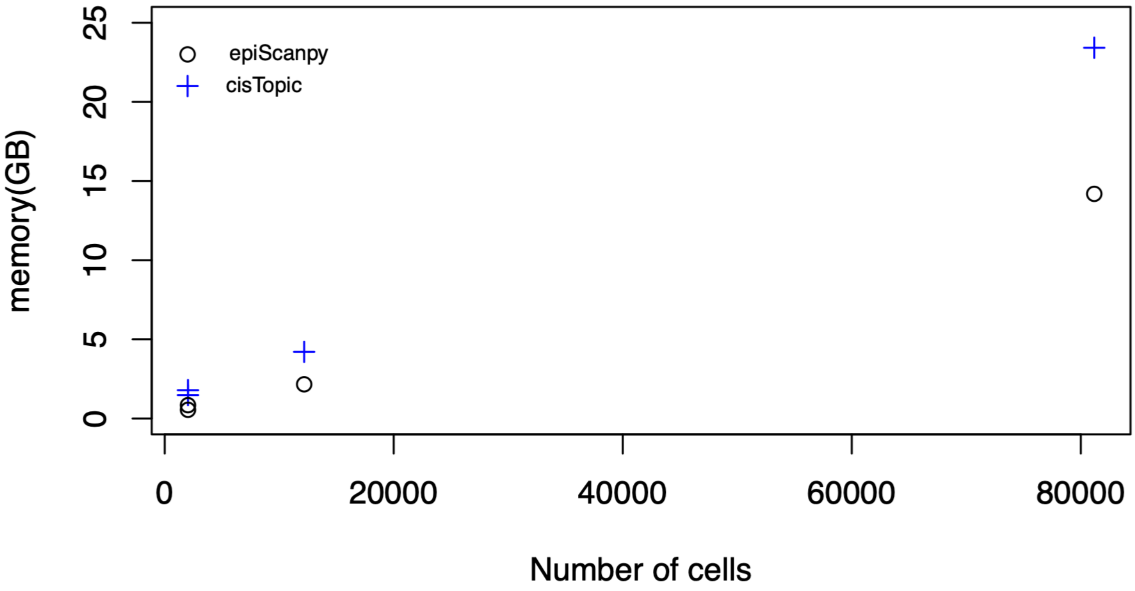

Suppl. Figure 13e: EpiScanpy and cisTopic performance: a) Runtime and **b)** memory usage for epiScanpy compared to cisTopic, running in CentOS Linux 7, on an AMD Opteron 6376 2.3GHz machine with 8 cores of CPU, 180GB of memory, and 245/45.2 MB/s of input/output speeds; comprising: reading input files, quality control, analysis, plotting and saving the results. The number of cells correspond to 2034 cells for the Buenrostro et al. dataset, 12178 for the subset Cusanovich et al. dataset, and 81173 for the full Cusanovich et al. dataset. On the full Cusanovich et al. dataset, CisTopic could not finish data matrix construction using the function createcisTopicObjectFromBAM, as it had reached the memory limit of 233 GB after 62.94 hours and was killed. To bypass matrix construction using cisTopic, we did input the count matrix generated by epiScanpy for the same dataset as a sparse matrix to cisTopic using the R package Seurat v3 (https://www.cell.com/cell/fulltext/S0092-8674(19)30559-8) and using the function createcisTopicObject.
