# Supplementary material for: EpiScanpy: integrated single-cell epigenomic analysis
Source: Nat Commun. 2021 Sep 1;12:5228. doi: 10.1038/s41467-021-25131-3 (PMC8410937; doi:10.1038/s41467-021-25131-3)
Supplement: Supplementary file 2 — Reporting Summary [file 41467_2021_25131_MOESM2_ESM.pdf]

nature research

Corresponding author(s): Maria Colome-Tatche and Fabian J. theis

Last updated by author(s): Jan 29, 2021

Reporting Summary

Nature Research wishes to improve the reproducibility of the work that we publish. This form provides structure for consistency and transparency in reporting. For further information on Nature Research policies, see our Editorial Policies and the Editorial Policy Checklist.

Statistics

For all statistical analyses, confirm that the following items are present in the figure legend, table legend, main text, or Methods section.

n/a	Confirmed	
☐	☒	The exact sample size (*n*) for each experimental group/condition, given as a discrete number and unit of measurement
☒	☐	A statement on whether measurements were taken from distinct samples or whether the same sample was measured repeatedly
☒	☐	The statistical test(s) used AND whether they are one- or two-sided<br>*Only common tests should be described solely by name; describe more complex techniques in the Methods section.*
☐	☒	A description of all covariates tested
☐	☒	A description of any assumptions or corrections, such as tests of normality and adjustment for multiple comparisons
☒	☐	A full description of the statistical parameters including central tendency (e.g. means) or other basic estimates (e.g. regression coefficient) AND variation (e.g. standard deviation) or associated estimates of uncertainty (e.g. confidence intervals)
☒	☐	For null hypothesis testing, the test statistic (e.g. *F*, *t*, *r*) with confidence intervals, effect sizes, degrees of freedom and *P* value noted<br>*Give P values as exact values whenever suitable.*
☒	☐	For Bayesian analysis, information on the choice of priors and Markov chain Monte Carlo settings
☒	☐	For hierarchical and complex designs, identification of the appropriate level for tests and full reporting of outcomes
☒	☐	Estimates of effect sizes (e.g. Cohen's *d*, Pearson's *r*), indicating how they were calculated

Our web collection on statistics for biologists contains articles on many of the points above.

Software and code

Policy information about availability of computer code

Data collection	All the data used was already publicly available and downloaded without the use of any software
Data analysis	Please ensure all the data analysis software/packages mentioned in the manuscript are listed in this reporting summary (with version numbers). For example: R version 3.6.1, cellranger v1.2.0, etc.<br>- For peak calling we used MACS2 (2.2.6 & 2.2.7)<br>- For the benchmarking of epiScanpy performances, we installed cisTopic v0.3.0, obtained from https://github.com/aertslab/cisTopic, and Seurat v3.2.3, running on R v3.6.1.<br>- For the processing of single cell data produced using the 10X technology, we used cellranger v1.2.0<br>- For the data analysis we used: Python (v3.6.0 & 3.7.9) , Scanpy (v1.6.0, v1.4.1 & 1.5.1, scanpy==1.4.5.2.dev6+gfa408dc7), AnnData (v0.7.4 & 0.6.22), epiScanpy (v0.2.2, 0.3.0 https://github.com/colomemaria/episcanpy-paper), seaborn (v0.9.0 , 0.11.0 & 0.11.1)

For manuscripts utilizing custom algorithms or software that are central to the research but not yet described in published literature, software must be made available to editors and reviewers. We strongly encourage code deposition in a community repository (e.g. GitHub). See the Nature Research guidelines for submitting code & software for further information.

Data

Policy information about availability of data

All manuscripts must include a data availability statement. This statement should provide the following information, where applicable:

- Accession codes, unique identifiers, or web links for publicly available datasets
- A list of figures that have associated raw data
- A description of any restrictions on data availability

Please provide a complete data availability statement here in the reporting summary, as provided in the manuscript.

Methylation data
- methylation profiles from Luo et al. 2017 available on Gene Expression Omnibus (GEO), accession number : GSE97179 https://www.ncbi.nlm.nih.gov/geo/query/acc.cgi?acc=GSE97179

Chromatin data
- brain chromatin accessibility datasets from Fang et al. 2019 available at http://renlab.sdsc.edu/r3fang/share/
- brain 10x datasets, downloaded from http://renlab.sdsc.edu/r3fang/share/ as 5kb size windows count matrix.
- scATAC-seq 10x PBMC 10k cells, obtained using the 10x chemistry Next Gem v1.1, and analysed using cellranger v1.2.0. The fragment files downloaded had been aligned on hg19. Data available here: https://cf.10xgenomics.com/samples/cell-atac/1.2.0/atac_pbmc_10k_nextgem/
-Satpathy et al.12 whole blood fresh data: The raw peak count matrix as well as the fragment files from the corresponding samples were downloaded from GEO: GSE129785. GSE129785_scATAC-Hematopoiesis-All.mtx.gz https://www.ncbi.nlm.nih.gov/geo/query/acc.cgi?acc=GSE129785
-Buenrostro et al. 201813 PBMC scATACseq, and Cusanovich et al. 201814: these datasets were used for the benchmarking of epiScanpy using the framework from Chen et al.15. They were downloaded from https://github.com/pinellolab/scATAC-benchmarking.

Genome annotations:
- promoter annotation was obtained from the Eukaryotic Promoter Database at https://epd.epfl.ch/
- enhancer annotation was obtained from the Mouse Encode Project at Ren Lab, http://chromosome.sdsc.edu/mouse/download.html
- gene annotation was obtained from UCSC and gencode https://www.gencodegenes.org/human/ using GRCh38.p13 for human data (ftp://hgdownload.soe.ucsc.edu/goldenPath/hg38/) and GRCm38.p6 for mouse data (ftp://hgdownload.soe.ucsc.edu/goldenPath/mm10/)
- Peaks: peaks used to analyse the Satpathy et al. data are available from GEO. GSE129785 https://www.ncbi.nlm.nih.gov/geo/query/acc.cgi?acc=GSE129785
Peaks used to build the count matrices for 10x 10k PBMC data were called using MACS2. A merge set of peaks was used to integrate the Satpathy et al. and the 10x 10k PBMC data, using the union of the aforementioned peaks.

Field-specific reporting

Please select the one below that is the best fit for your research. If you are not sure, read the appropriate sections before making your selection.

☒ Life sciences ☐ Behavioural & social sciences ☐ Ecological, evolutionary & environmental sciences

For a reference copy of the document with all sections, see nature.com/documents/nr-reporting-summary-flat.pdf

Life sciences study design

All studies must disclose on these points even when the disclosure is negative.

Sample size	No dataset has been produced in this paper. We chose our samples from publicly available datasets containing single-cell chromatin sequencing data and DNA methylation data
Data exclusions	No data have been excluded from the analysis. We used whole samples from relevant tissues of publicly available datasets
Replication	No dataset has been produced in this paper. The data analysis can be replicated using https://github.com/colomemaria/episcanpy-paper
Randomization	No dataset has been produced in this paper.
Blinding	No dataset has been produced in this paper. From the publicly available data used, none contain different experimental groups that required blinding. Moreover, when multiple samples were used we checked and eventually corrected for batch effect.

Reporting for specific materials, systems and methods

We require information from authors about some types of materials, experimental systems and methods used in many studies. Here, indicate whether each material, system or method listed is relevant to your study. If you are not sure if a list item applies to your research, read the appropriate section before selecting a response.

Materials & experimental systems

n/a	Involved in the study
☒	☐ Antibodies
☒	☐ Eukaryotic cell lines
☒	☐ Palaeontology and archaeology
☒	☐ Animals and other organisms
☒	☐ Human research participants
☒	☐ Clinical data
☒	☐ Dual use research of concern

Methods

n/a	Involved in the study
☒	☐ ChIP-seq
☒	☐ Flow cytometry
☒	☐ MRI-based neuroimaging
